# SPEAR: CRISPR-mediated ultrasensitive, specific and rapid one-pot detection strategy for cancer-related SNPs

**DOI:** 10.7150/thno.107488

**Published:** 2025-02-18

**Authors:** Linlin Bai, Yanan Pang, Ting Wang, Shengzhou Wang, Kaiming Guo, Tian Xuan, Ziqin Zhang, Dianwei Liu, Feng Qian, Yan Zheng, Gang Jin, Rui Wang

**Affiliations:** 1 Center for Medical Research and Innovation, Shanghai Pudong Hospital, Human Phenome Institute, State Key Laboratory of Genetic Engineering, School of Life Sciences, School of Pharmacy, Fudan University, Shanghai 200438, China.; 2 Department of Gastroenterology, Shanghai Institute of Pancreatic Diseases, Changhai Hospital; National Key Laboratory of Immunity and Inflammation, Naval Medical University, Shanghai 200433, China.; 3 Department of Hematology, Peking University Shenzhen Hospital, Shenzhen Peking University-The Hong Kong University of Science and Technology Medical Center, Shenzhen 518036, China.; 4 College of Biological Science and Engineering, Fuzhou University, Fuzhou 350108, China.; 5 Department of Hepatobiliary Pancreatic Surgery, Changhai Hospital, Naval Medical University, Shanghai 200433, China.; 6 International Human Phenome Institutes, Shanghai 200433, China.

**Keywords:** CRISPR, NEAR, one-pot, SNP detection, cancer screening

## Abstract

**Rationale:** The ultrasensitive and accurate detection of driver mutations is critical for early cancer screening and precision medicine. Current methods face challenges in balancing sensitivity, specificity, and speed, which limits their clinical utility. Therefore, a rapid, sensitive, and specific method is essential for detecting cancer-related SNPs.

**Methods:** This study introduces SPEAR (Specific Point mutation Evaluation via CRISPR-Cas Assisted Recognition), a novel methodology combining NEAR (Nicking Enzyme Amplification Reaction) isothermal amplification with SNP-specific recognition by Cas12b RNP in a one-pot configuration to detect cancer-related single nucleotide polymorphisms (SNPs). SPEAR leverages the power of NEAR isothermal amplification to efficiently amplify target DNA, followed by Cas12b RNP for SNP-specific recognition. This integrated approach ensures a rapid and precise mutation detection system in a single reaction.

**Results:** The method was applied to blood samples for the detection of cancer-related mutations, with results obtained in approximately 30 min. The SPEAR enables detection of gene mutations at the single-molecule level and it can detect targets at a 0.1% ratio despite strong background interference. The method exhibits single-base resolution specificity, allowing for the detection of multiple SNPs in a single reaction. It outperforms first-generation sequencing (FGS) in both convenience and sensitivity, while remaining compatible with next-generation sequencing (NGS).

**Conclusion:** SPEAR offers a rapid, sensitive, and convenient approach to detect cancer-related SNPs, with significant potential for clinical applications, including real-time detection and molecular diagnostics in precision medicine.

## Introduction

Improvements of molecular genetics, genome sequencing technologies and bioinformatics highlight the crucial role of genetic testing in precision oncology for auxiliary diagnosis, therapy selection, and cancer recurrence monitoring 1-3. Emerging research increasingly links single nucleotide polymorphisms (SNPs) to cancer initiation and progression, emphasizing the need for highly sensitive and accurate SNPs detection 4, 5. For instance, SNPs occurring at the 12^th^ codon of *KRAS* gene usually appeared in various cancers, which accounts for 88.8% of pancreatic cancer (PC) mutation cases 6. These imply that we need to develop convenient strategies for the detection of cancer-related SNPs.

Existing SNP detection techniques include amplification refractory mutation system-polymerase chain reaction (ARMS-PCR), droplet digital PCR (ddPCR), first-generation sequencing (FGS), and next-generation sequencing (NGS). However, these methods are still limited for the clinical SNPs detection. To be specific, both FGS and NGS require expensive sequencing equipment, complex operational procedures, and specialized personnel, making them difficult to implement widely in resource-limited settings. In addition, the limit of detection (LOD) of FGS is 10% to 20%, which is insufficient to identify low-abundance mutations 7. Meanwhile, the turnaround time of NGS is long as one week, which is not suitable for clinical decision-making. The entire process of ARMS-PCR and ddPCR typically takes between 2 to 4 hours and they are widely used 8-10. But expensive equipment is also required, and it is difficult to detect multiple mutations at the same point simultaneously with a single assay due to cross interference between probes 11. Therefore, it is urgent to develop a sensitive, rapid, cost-effective, and convenient method that could detect multiple cancer-related SNPs simultaneously.

Currently, next-generation molecular diagnostic techniques based on clustered regularly spaced short palindromic repeats (CRISPR) are attracting much attention due to their remarkable sensitivity and specificity 12, 13. Including Cas12 and Cas13, many CRISPR-associated (Cas) proteins have both cis-cleavage and trans-cleavage activities 14-16. Single guide RNA (sgRNA) combines with Cas12 to form a ribonucleoprotein complex capable of recognizing the protospacer adjacent motif (PAM). Upon heteroduplex formation with the sgRNA, the target DNA strand undergoes cleavage by the RuvC domain. The active site of RuvC remains accessible, enabling the trans-cleavage of non-target single-stranded DNA (ssDNA) after the release of the PAM-distal cleavage product 16, 17. By incorporating ssDNA labeled with fluorescent and quencher molecules into the CRISPR reaction, a fluorescent signal would be generated, enabling visual detection of the target. However, the equilibrium dissociation constants of Cas proteins for their targets are in the picomolar concentration range (10^-10^ to 10^-12^ M) 16, 18, hence combining CRISPR with nucleic acid amplification methods becomes a suitable choice for achieving sensitive detection. Nevertheless, due to the incompatibility between nucleic acid amplification and CRISPR systems, two-step reaction is often required, which is complicated and increasing the risk of amplicon contamination 19-21. While some one-pot CRISPR methods have been developed, they are inconvenient in terms of performance, require long reaction time, and are unable to detect multiple mutations in a single reaction 22, 23. Therefore, a simple CRISPR-based one-pot method with high sensitivity and simplified reaction components is still needed for cancer-related SNPs detection.

Here, we developed a novel one-pot CRISPR-based method, which is termed SPEAR (Specific Point mutation Evaluation via CRISPR-Cas Assisted Recognition), for the convenient, sensitive, and specific detection of cancer-related SNPs. In our SPEAR assay, it could detect SNPs at single-molecule level and identify targets at a 0.1% ratio from strong background interference. Notably, SPEAR contains sgRNAs with a strong ability to recognize single-base mutations and no cross-interference, enabling for the detection of multiple SNPs within a single reaction. The SPEAR method is characterized by its simplicity, involving the rapid release of nucleic acids from blood samples, with the entire process typically completed in approximately 30 min. To evaluate its clinical applicability, we applied the SPEAR method to analyze 107 clinical samples, comparing it with sequencing methods, further showed its practicality in clinical settings. SPEAR is more convenient and sensitive than FGS, while maintaining compatibility with NGS, making it highly promising for point-of-care cancer screening and precision medicine applications.

## Materials and methods

### Materials and reagents

Nt.BstNBI, Bst 3.0 DNA polymerase, and Fluorescent Dye were obtained from New England Biolabs (MA, USA). Recombinant RNase Inhibitor, TaKaLa Taq™ Hot Start Version, 10 × gel loading buffer was obtained from Takara (Dalian, China). The dNTP mixture was purchased from Vazyme (Nanjing, China). KOD Fx was provided by TOYOBO (Shanghai, China). TIANgel Midi Purification Kit, Serum/Plasma Circulating DNA Kit and TIANamp Genomic DNA Kit were procured from Tiangen Biotech Co., Ltd. (Beijing, China). HiPure RNA Pure Micro Kit was provided by Magen (Guangzhou, China). Fetal bovine serum (FBS) and Dulbecco's Modified Eagle's Medium (DMEM) were bought from Thermo Fisher Scientific (MD, USA). The TBE buffer and additional chemical reagents were supplied by Sangon Biotech (Shanghai, China). The AapCas12b was supplied by Tolobio (Shanghai, China). All oligonucleotide sequences and ssDNA reporter were synthesized by Sangon Biotech (Shanghai, China).

All clinical samples were kindly provided by Changhai Hospital (Shanghai, China). Clinical samples for this study were obtained from patients who gave informed (oral) consent, and the study protocol received approval from the Scientific Research Ethics Review Committee of Changhai Hospital.

### Nucleic acid extraction of blood samples

Standard nucleic acid extraction from blood samples with commercial kit, was according to instruction manual. Briefly, fresh blood samples were collected using EDTA anticoagulant tubes, and plasma was separated by centrifugation at 3000 rpm for 10 min. The plasma was then extracted using the TIANGEN Serum/Plasma Circulating DNA Kit. Finally, 50 μL DNA was collected and stored at -80 °C for further use.

For rapid nucleic acid extraction from blood samples, 500 µL blood sample was treated with 1500 µL red blood cell lysis buffer at room temperature for 1 min, and the cell precipitate was obtained by 30 s 12000 rpm centrifugation. Then, DNA was extracted from the cell precipitate by treating it with 100 µL of nucleic acid releaser at a temperature of 95 °C for 3 minutes. 2 µL of obtained cell lysate was directly used as template for SPEAR assay.

### Cas12b-based cleavage

The 20 μL Cas12b-mediated nucleic acid cleavage system contained 1 × Tolo buffer, 4 U RNase inhibitor, 0.15 μM AapCas12b, 0.3 μM sgRNA, 1 μM ssDNA reporter, and 100 nM double-stranded DNA template. The reaction system was heated at 57 °C for 30 min, and fluorescence was monitored in real-time using the CG real-time fluorescence quantitative PCR instrument (Jingle, Hangzhou, China). After completing the reaction, the tube was immediately placed under blue light (Monad, Suzhou, China), and the fluorescence results were photographed with a smartphone.

### SPEAR assay

SPEAR system was prepared by combining 4.8 μL of Part I and 20.2 μL of Part II. Part I consisted of primers (10 µM F and 10 µM R primers, each 1 µL), dNTP mixture (10 mM, 0.8 µL) and DNA template (2 µL)). Part II consisted of Nt.BstNBI (10 U/µL, 1 µL), Bst 3.0 DNA polymerase (8 U/µL, 0.6 µL), Isothermal Amplification Buffer II Pack (10 ×, 1.25 µL), Tris-HCl (pH 7.9) (250 mM, 1.25 µL), NaCl (500 mM, 1.25 µL), BSA (500 µg/mL, 1.25 µL), AapCas12b (10 µM, 0.4 µL), sgRNA (10 µM, 1 µL for each), HOLMES Buffer 1 (10 ×, 2 µL), Recombinant RNase Inhibitor (4 U/µL, 1.25 µL), ssDNA reporter (20 µM, 1.25 µL), add DEPC treated water to 20.2 µL. Part I and Part II were mixed and then incubated at 57 °C for 30 min in real-time fluorescence PCR system. Real-time fluorescence was monitored with 1 min intervals.

### qPCR amplification

The 50 μL PCR amplification system was composed of TaKaRa Taq HS (5 U/μL, 0.25 μL), dNTP mixture (each 2.5 mM, 4 μL), PCR buffer (including 10 mM Tris HCl pH 8.3, 50 mM KCl, 1.5 mM MgCl_2_) (10 ×, 5 μL), forward and reverse primer (10 μM, each 2 μL), TaqMan probe (10 μM, 1 μL), target DNA (2 μL), and 32.75 μL DEPC treated water. PCR was performed with an initial 5 min hot start at 95 °C, followed by 40 cycles consisting of 20 s at 95 °C, 30 s at 55 °C, and 30 s at 72 °C, ending with a final extension at 72 °C for 5 min. The sequences for the qPCR system are provided in Table S4.

### Statistical analysis

All experiments were conducted in triplicate. Statistical analysis was performed using GraphPad Prism 8.0. An unpaired two-tailed Student's t-test was applied for comparisons between two groups. Data are presented as the mean ± standard error. value < 0.05 was considered statistically significant. “ns” indicates no significance.

## Results

### Rational design of SPEAR

**Figure 1:**
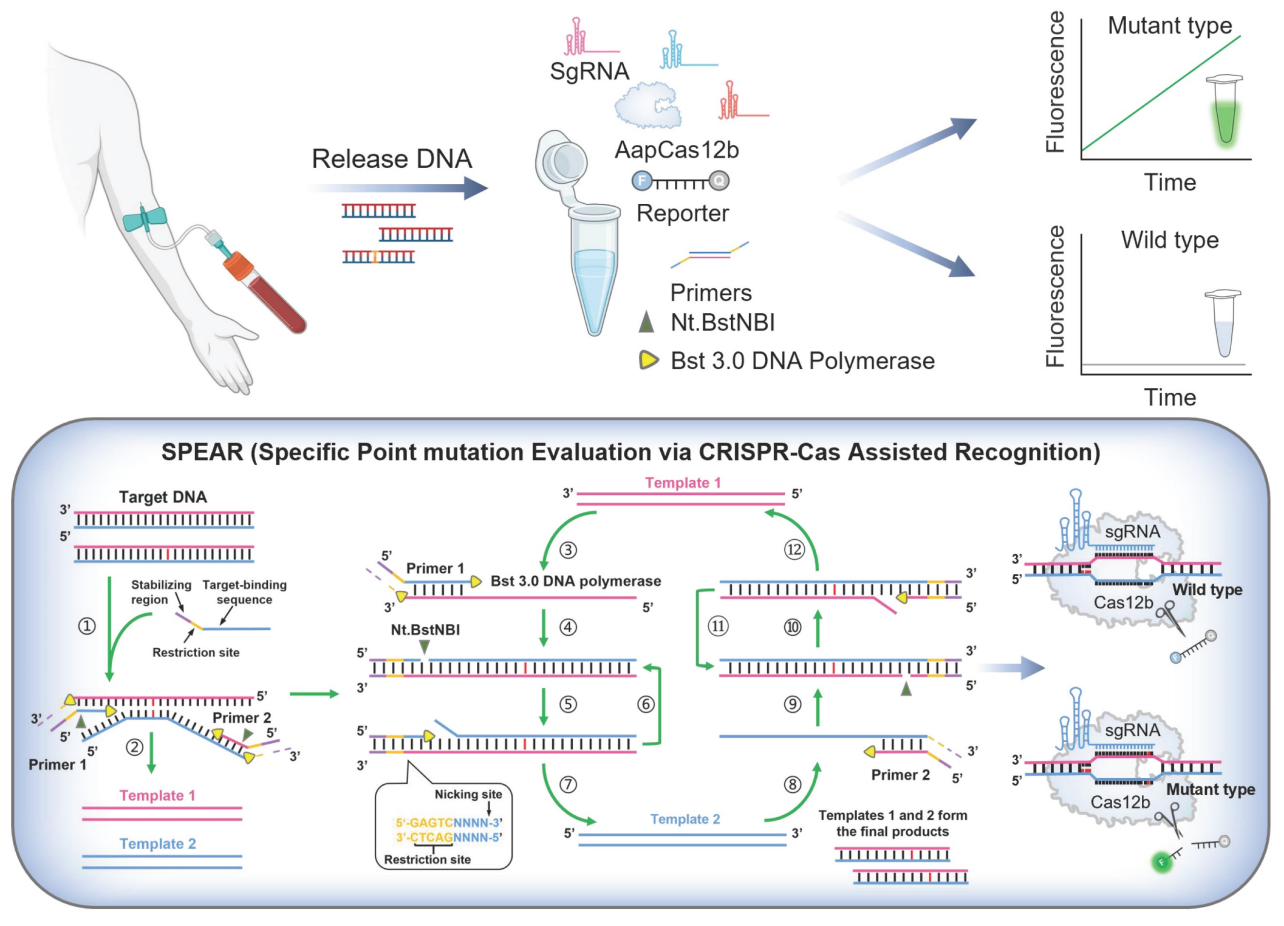
The scheme of one-pot CRISPR based SPEAR assay. DNA extracted from blood sample was used as template for SPEAR reaction. After 30 min incubation, fluorescence under blue light indicates the presence of SNPs, while the absence of fluorescence indicates no SNPs. For SPEAR principle, DNA polymerase with strong strand displacement activity opens the template double strand, and primers bind to the target DNA to initiate amplification. Polymerase extends primers, forming a dsDNA template that contains a recognition site for the nicking enzyme. While the nicking enzyme introduces nicks, the polymerase rebinds at the nick and extends the strand, thereby restoring the recognition site for the nicking enzyme and facilitating strand displacement. The continuous cycle of extension, cleavage, and strand displacement results in exponential amplification of the target DNA. Cas12b/sgRNA RNP targets mutant sequences, activating Cas12b to cleave the quenched ssDNA reporter, producing fluorescent signals. The WT sequences could not activate Cas12b and the reaction tube showed no fluorescence. Image created with BioRender.com, with permission.

The detection scheme of SPEAR is shown in Figure 1. DNA was extracted from the blood sample, serving as the template for the SPEAR assay. Following a 30 min reaction, fluorescence under blue light indicated the presence of cancer-related SNPs within the sample. In contrast, no fluorescence demonstrated the absence of related SNPs. The principle of SPEAR involves the following key steps: In the initial stage of the NEAR reaction, the target DNA double strands are opened, allowing primers to bind to the template. NEAR amplification then generates a large number of double-stranded DNA (dsDNA) products. Finally, Cas12b recognizes the dsDNA and releases a fluorescent signal. To be specific, (1) opening of target DNA and primer binding. Bst 3.0 DNA polymerase, with strong strand displacement activity, opens the target DNA double strand. The primer then binds to the template, and Bst 3.0 DNA polymerase extends along the 3' end of the primer. The 5' end of the primer contains a specific recognition site for the nicking enzyme. (2) Nicking by Nt.BstNBI endonuclease and polymerase extension generates a few single-stranded templates. The Nt.BstNBI endonuclease recognizes the restriction site (5'-GAGTCNNNN-3') and cleaves the single strand, creating a nick. The polymerase binds to the nicked DNA and and uses the uncut single strand as a template to extend the cut single strand, displacing the previously extended strand, thereby forming single-strand DNA templates 1 and 2. (3-4) Single-stranded template binds with primer, extends, and nicks to form double-stranded intermediate. Primer 1 binds to template 1, and Bst 3.0 DNA polymerase extends the primer to form double-stranded intermediate with a restriction enzyme recognition site. Nt.BstNBI endonuclease recognizes the site and cleaves the strand again, generating a new nick. (5-7) Repeated nicking and extension leading to accumulation of template 2. The polymerase binds to the new nick in the double-stranded intermediate and synthesizes a new double-stranded nucleic acid with a restored recognition region for the nicking enzyme, while the displaced old strand serves as template 2. This cycle of cutting, extending, and displacing repeats, generating numerous single-stranded DNA template 2. (8-12) Accumulation of template 1. Primer 2 binds to the generated single-stranded DNA template 2, repeating the processes of incising, extension, and displacement to generate a large number of single-stranded DNA template 1. Templates 1 and 2 hybridize to form a large number of dsDNA templates. Continuous template production leads to exponential amplification. During amplification, Cas12b/sgRNA ribonucleoprotein (RNP) specifically recognizes the mutant-type (MT) target, and activated Cas12b cleaves the quenched ssDNA reporter to generate fluorescent signals. However, when the sample contains only the wild-type (WT) template, Cas12b stays inactive and does not cleave the ssDNA reporter. The isothermal amplification and Cas12b cleavage occur at the same time; therefore, a truly one-pot CRISPR-based detection system has been established.

### Development of SPEAR

**Figure 2:**
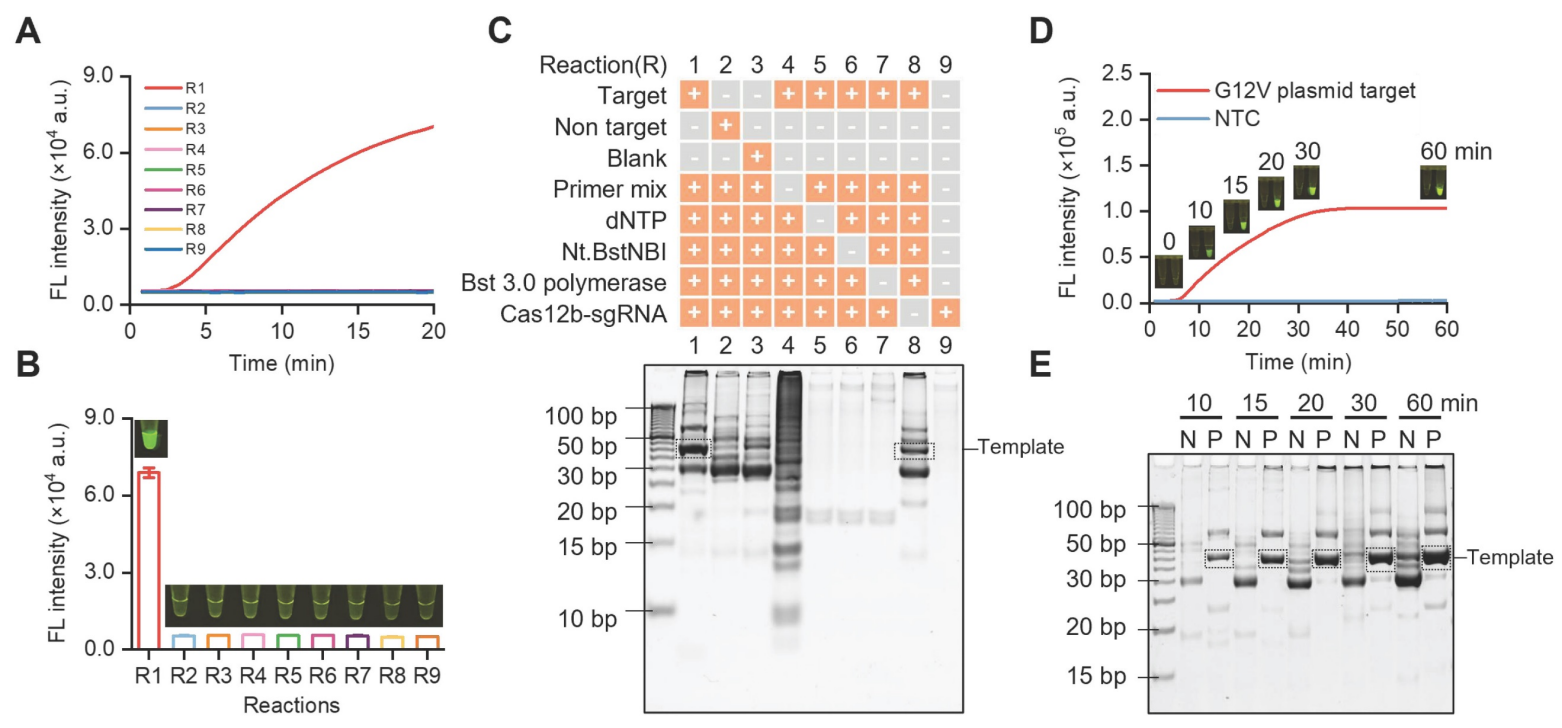
SPEAR system constructed with different components omitted (R1-R9). (A) Real-time fluorescence of the nine reaction systems. (B) End-point fluorescence and visual detection results of the nine reaction systems. (C) The products of nine reaction systems (R1-R9) analyzed by PAGE gel electrophoresis. (D) Real-time fluorescence curve aligned with fluorescence images at different time points of SPEAR reaction. NTC: no template control. (E) PAGE gel electrophoresis analysis of SPEAR products after different reaction times. N: no template control. P: 1e4 copies of G12V (c.35G>T) plasmid target. Data were the mean ± SD (n = 3).

For proof-of-concept demonstration, we used SPEAR to detect the *KRAS* gene mutation in pancreatic cancer. We synthesized a plasmid containing the G12V (c.35G>T) mutation as target and explored the feasibility of SPEAR. Specific primers and sgRNA were designed with a template length of 42 bp. To study the impact of each component on SPEAR reaction, nine reaction systems (R1-R9) were built up with different components omitted. Each reaction was carried out at 57 °C for 20 min. Real-time fluorescence and endpoint fluorescence were monitored, and the reaction products were further analyzed by PAGE electrophoresis. It was observed that only when all components of SPEAR existed, fluorescence would generate in the reaction tube (Figure 2A-B). In addition, the fluorescence generated immediately at the beginning when template concentration was 1e4 copies, demonstrating ultrahigh efficiency of SPEAR reaction. We further verified this result with gel electrophoresis. Results showed that reaction system with all components of SPEAR generated a 42 bp template fragment (Figure 2C). Additionally, gel imaging results showed that, although significant non-specific amplification existed, there was no fluorescence during visual detection, confirming the high specificity of the proposed SPEAR visual detection method. To investigate whether the accumulation of non-specific products leads to background fluorescence that interferes with result determination, we further tested the SPEAR reaction at different reaction times (Figure 2D). In short reaction time, there was a reduction in non-specific amplicons. With the extension of incubation time, the specific and nonspecific amplicons were both becoming thicker and making it hard to distinguish with gel electrophoresis (Figure 2E). Nevertheless, our proposed SPEAR visual detection method could achieve detection easily, with obvious fluorescence in positive samples and absolutely no fluorescence in negative ones. Moreover, for samples with 1e4 copies of templates, clear fluorescence was generated after only a 10 min reaction. Therefore, it demonstrated high specificity and efficiency of the proposed SPEAR method.

### Specificity study of SPEAR for single base mutation differentiation and cancer-related SNPs detection system built up

**Figure 3:**
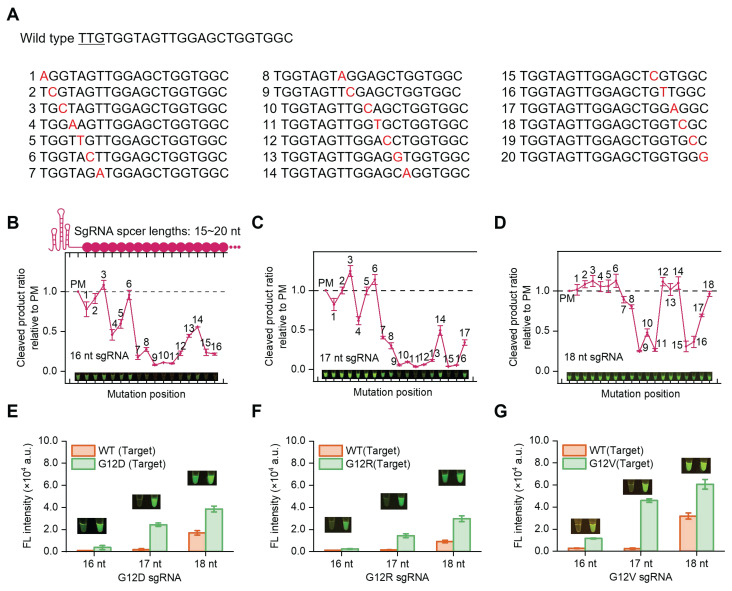
Specificity evaluation of varying lengths of sgRNAs on single-base mutation templates. (A) The WT spacer sequence contains the *KRAS* G12 gene and the corresponding gene sequence with mismatch sites. The ratio of endpoint fluorescence values between mismatched dsDNA and perfectly matched (PM) dsDNA templates with sgRNA spacer lengths of (B) 16 nt, (C) 17 nt, (D) 18 nt. Corresponding visualization results were also presented. Specificity evaluation of SPEAR for detecting G12D (c.35G>A) (E), G12R (c.34G>C) (F), and G12V (c.35G>T) (G) with different lengths of sgRNAs (16-18 nt), respectively. The 30-min endpoint fluorescence values and visualization results were presented. Data were the mean ± SD (n = 3).

In SPEAR reaction, a mutation-specific sgRNA is required to avoid the cross-reactivity against WT. Different lengths of sgRNAs with single base mismatch templates were synthesized to determine the specificity of Cas12b in SNP detection (Figure 3A). Briefly, the cocktail containing AapCas12b, sgRNA, ssDNA reporter, and dsDNA template was incubated at 57 °C for 30 min. The ssDNA reporter was cleaved by activated Cas12b and generated fluorescence signals after the sgRNA bound complementarily to the templates. Results indicated that the 16 nt sgRNA was unable to tolerate single mismatches at position 7-16, except at position 14. The 17 nt sgRNA was unable to tolerate single mismatches at position 9-16, except at position 14. The 18 nt sgRNA showed no tolerance for single mismatches at positions 9, 10, 11, 15 and 16 (Figure 3B-D). In contrast, 19-20 nt sgRNAs exhibited tolerance to single-base mismatches. The 15 nt sgRNA could identify single-base mutations at positions 7-15, except at position 14; however, it showed low reaction efficiency (Figure S1). Accordingly, sgRNAs with three different lengths (16-18 nt) were designed to detect pancreatic cancer hotspot mutation G12V (c.35G>T), G12D (c.35G>A), G12R (c.34G>C), respectively (Figure 3E-G). The results indicated that 16 nt and 17 nt sgRNAs could differentiate the mutation from WT, with the 17 nt sgRNA generating higher fluorescence intensity. Therefore, the 17 nt sgRNA was chosen for detecting the *KRAS* G12V (c.35G>T), G12D (c.35G>A) and G12R (c.34G>C) mutations.

### Reaction components and conditions optimization of SPEAR

**Figure 4:**
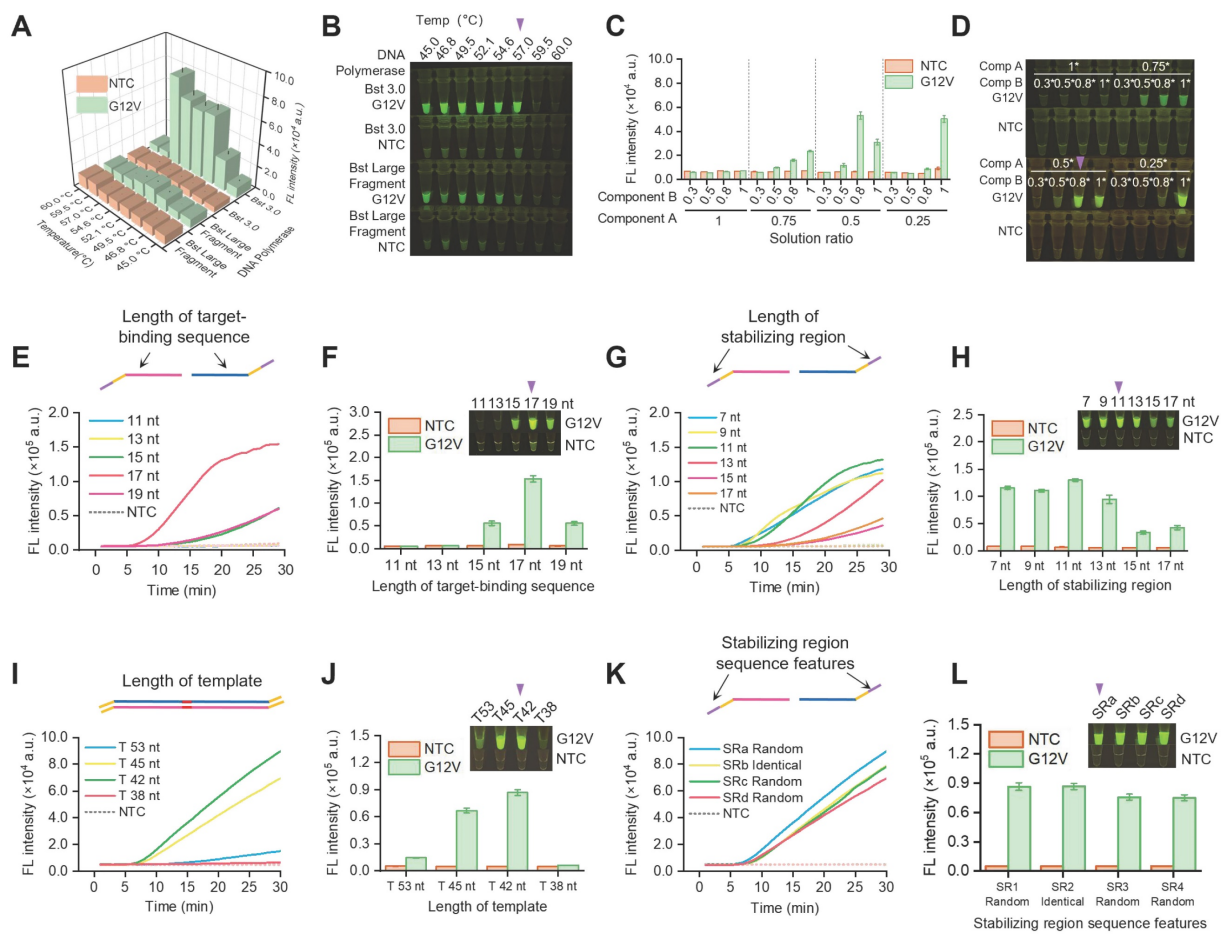
Optimization of reaction components and conditions for SPEAR. Study of polymerase, temperature and reaction component in SPEAR system. (A), (B) Bst 3.0 DNA polymerase and Bst DNA large fragment polymerase were employed for SPEAR reaction under different temperature conditions. The 30-min endpoint fluorescence values and visualization results were presented. (C), (D) Establishment of the SPEAR ionic system by optimizing the ratio between component A and component B in the integrated reaction. Component A contained the NEAR amplification components and component B included the Cas12b cleavage components. In a 25 µL total reaction volume, 0.5 × component A consists of: Nt.BstNBI (10 U/µL, 1 µL), Bst 3.0 DNA polymerase (8 U/µL, 0.6 µL), Isothermal Amplification Buffer II Pack (10 ×, 1.25 µL), Tris-HCl (pH 7.9, 250 mM, 1.25 µL), NaCl (500 mM, 1.25 µL), and BSA (500 µg/mL, 1.25 µL). 0.8 × component B consists of: AapCas12b (10 µM, 0.4 µL), sgRNA (10 µM, 1 µL), HOLMES Buffer 1 (10 ×, 2 µL), Recombinant RNase Inhibitor (4 U/µL, 1.25 µL), and ssDNA reporter (20 µM, 1.25 µL). The 30-min endpoint fluorescence values and visualization results were presented. (E)-(J) Primer design in the SPEAR system. Optimization of the length of the stabilizing region, target-binding sequence and template in the primer. (K), (L) Investigation of the sequence features of stabilizing region. SRa, SRb, SRc, SRd were four pairs of primers designed for SPEAR reaction. Among them, the stabilizing regions of the forward (F) and reverse (R) primers for SRa, SRc, and SRd were randomly designed to be different, while those of SRb were designed to be identical. The sequence information is shown in Table S3. The real-time fluorescence, 30-min endpoint fluorescence values, and visualization results were presented. The reactions (A) to (L) were performed using *KRAS* G12V (c.35G>T) 17 nt sgRNA and 1e4 copies of *KRAS* G12V (c.35G>T) plasmids as templates. The optimal conditions are marked by a purple triangle in the figure.

To reduce the whole detection time, we optimized the one-pot SPEAR system. Firstly, we optimized the polymerase and reaction temperature. The findings suggested that Bst 3.0 DNA Polymerase exhibited higher activity compared to Large Fragment Bst DNA Polymerase. Considering the optimal reaction temperature for the nicking enzyme is 55 °C, we optimized the reaction temperature in the range of 50-60 °C, and results indicated that 57 °C was best for one-pot reaction (Figure 4A-B). Therefore, 57 °C was chosen for one-pot reaction in the following assay. In the one-pot SPEAR system, component A was for NEAR and component B was for CRISPR/Cas12b cleavage reaction. The balance of component A and B was essential for effective one-pot detection, hence we studied the different proportional combination of component A and B (Figure 4C-D). When component A was at 1 ×, there was no fluorescence inside tube, demonstrating it greatly inhibited Cas12b cleavage. When component A was at 0.5 ×, there was bright florescence inside the tube when component B was at 1 × or 0.8 ×, demonstrating good amplification and Cas12b cleavage efficiency. When component A was reduced to 0.25 ×, good detection efficiency was demonstrated only when component B was at 1 ×. Based on the results, components A and B showed a wide range of proportional combinations for the one-pot SPEAR reaction. Ultimately, we selected 0.5 × component A and 0.8 × component B for the one-pot SPEAR reaction. Then we optimized the primer for one-pot SPEAR reaction. The primer contains a restriction site (5'-GAGTCNNNN-3') in the middle, a stabilizing region at 5' end, and a target-binding sequence at 3' end (Figure S2). The target-binding sequence at 3' end is essential for primer annealing to the template. We fixed the 5' end length at 11 nt and studied the 3' end length ranging from 11 nt~19 nt. Results indicated that with the 3' end length increasing from 11 nt to 19 nt, the fluorescence intensity gradually increased and reached the highest when the length was 17 nt (Figure 4E-F). Then, we fixed the 3' end length at 17 nt and studied the 5' end length from 7 nt to 17 nt. Results showed that from 7 nt to 13 nt, it could effectively amplify the target. When the length of the 5' end was too long, it would generate more unspecific amplicons and less specific amplicons that would be identified by Cas12b (Figure 4G-H). Therefore, we designed primer with 17 nt as 3' end and 11 nt as 5' end for SPEAR reaction. Next, we studied the impact of template length on the SPEAR reaction. Results indicated that with template length increased from 30 bp to 60 bp, the work efficiency of SPEAR initially increased and then decreased subsequently, with highest detection efficiency of 42 bp template length (Figure 4I-J). We assume that a template that is too short encounters steric effects from enzymes, while a template that is too long would generate more unspecific amplification. Therefore, we chose 42 bp as template length for SPEAR detection. Furthermore, we studied the oligonucleotide sequence of primer at 5' end. We designed 4 pairs of primers, with one pair having the same 5' end and the other three pairs designed randomly (Table S3). Results indicated that all primers could successfully amplify the target (Figure 4K-L). This discovery greatly simplified primer design by selecting only 17 nt sequence near the target detection region as the target-binding sequence of the primer (Figure S3). Additionally, the introduction of a restriction site makes it suitable for arbitrary sequence detection, whether or not the original target sequence contains a restriction site, thus greatly extending the application scope of the SPEAR method.

### Evaluation of sensitivity and specificity of SPEAR

**Figure 5:**
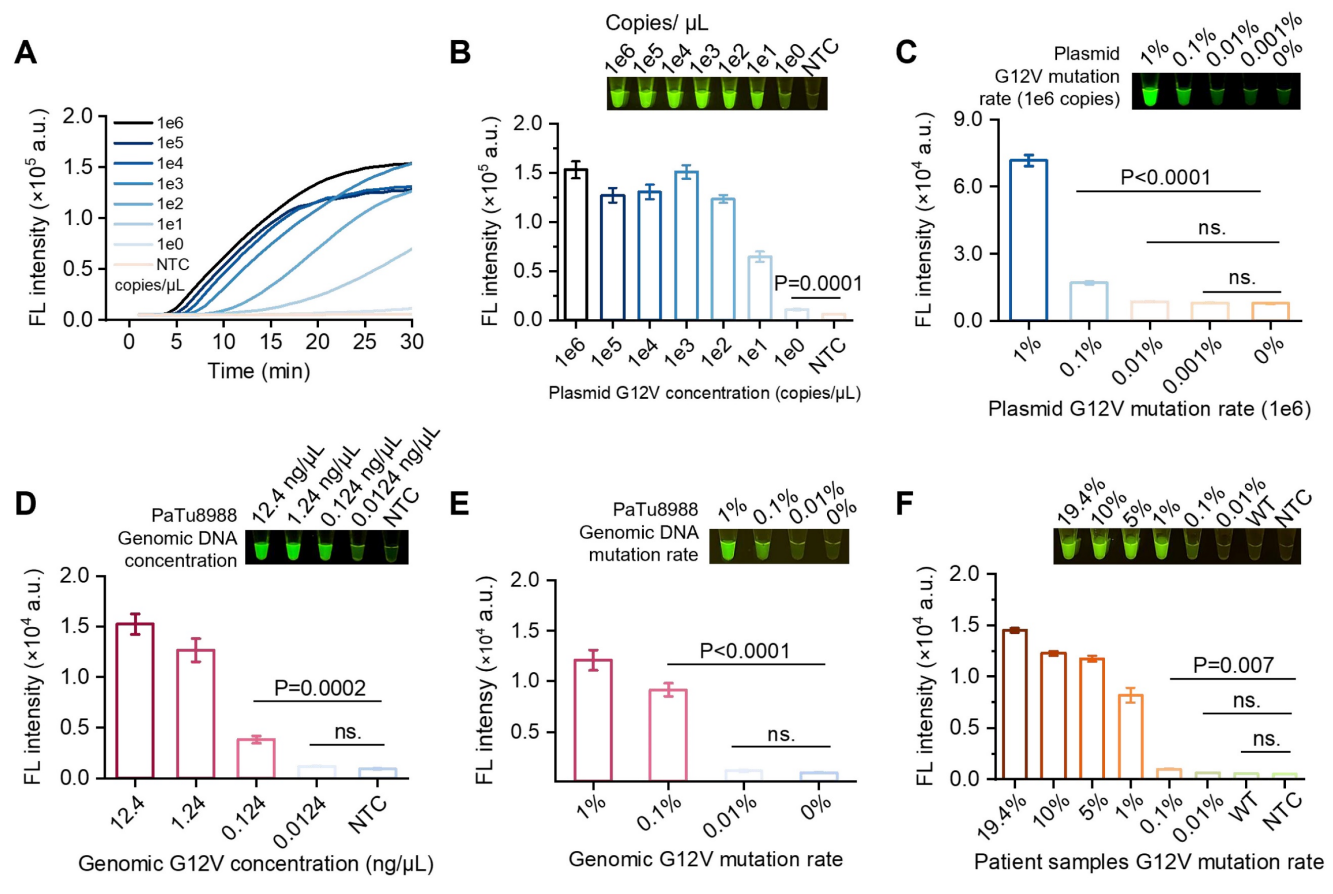
Evaluation of sensitivity and single-nucleotide mutation discrimination of SPEAR. (A) Real-time fluorescence of one-pot SPEAR reaction with *KRAS* G12V (c.35G>T) plasmid DNA subjected to 10-fold gradient dilution as templates. (B) The 30-min endpoint fluorescence values and visualization results. (C) Sensitivity of SPEAR method in detecting 1e6 copies of plasmid templates with gradient *KRAS* G12V (c.35G>T) mutation rates. (D) Detection sensitivity of genomic DNA from PaTu8988 cell line with SPEAR reaction at 57 °C for 30 min. (E) Sensitivity of mutation rate detection with genomic DNA as template. A mixture of PaTu8988 and 293T genomic DNA, totaling 80 ng and containing gradient *KRAS* G12V (c.35G>T) mutation rates, was used as templates for SPEAR detection. (F) Sensitivity of mutation rate detection with cfDNA as template. A mixture of cfDNA extracted from pancreatic cancer patients and healthy individuals, totaling 80 ng and containing gradient *KRAS* G12V (c.35G>T) mutation rates, was used as templates for SPEAR detection. Data were the mean ± SD (n = 3), P values were determined by two-tailed Student's t-tests. NTC: No template control.

The sensitivity and specificity of SPEAR method were initially evaluated by detecting plasmid. A 10-fold gradual dilution series of *KRAS* G12V (c.35G>T) plasmid was used as templates and SPEAR method was performed. Results showed the LOD of SPEAR was 1 copy per reaction (Figure 5A-B). As is known, in early cancer stage, the mutation accumulation is rare, the majority of DNA are WT sequences, which significantly interferes with the analysis. To test the feasibility of SPEAR method for rare mutation detection from high background interference, we mixed the MT and WT plasmids at various ratios, with G12V (c.35G>T) plasmid comprised 1, 0.1, 0.01, and 0.001% of the whole templates (1e6 copies), finding that the detection limit of G12V (c.35G>T) mutation was 0.1% (Figure 5C, S4A). Furthermore, we employed human genomic DNA extracted from cell lines as a template to evaluate the feasibility of SPEAR method. Results showed SPEAR method was able to detect 0.124 ng/µL, equivalent to 1.56 aM and 20 copies of human genomic DNA per reaction (Figure 5D, S4B). One step further, we mixed the genome DNA of two cell lines as mock DNA for SNP testing. The background was genomic DNA from the human normal hepatocyte cell line 293T, and the target was genomic DNA from the human pancreatic cancer cell line PaTu8988, which carries the G12V (c.35G>T) mutation. Mock DNA templates were prepared by adding mutant type cell line (MT, PaTu8988) to wild type cell line (WT, 293T) in a ratio ranging from 0.01% to 1% and tested by SPEAR method. Results showed the SPEAR method was able to detect *KRAS* G12V (c.35G>T) mutation down to 0.1% in high background interference of WT genomic DNA (Figure 5E, S4C). To simulate real-world clinical test scenario, we applied the SPEAR assay to cell free DNA (cfDNA) sample extracted from the plasma of pancreatic cancer patient. The sample contained *KRAS* G12V (c.35G>T) mutation at rate of 19.4% (Figure S5). We generated mutant-to-WT ratios ranging from 10% to 0.01% by mixing DNA from the pancreatic cancer patient with cfDNA from healthy individuals. The SPEAR system successfully realized the detection of mixed samples with LOD as low as 0.1% (Figure 5F, S4D). We performed a logarithmic analysis of the G12V (c.35G>T) mutation rate in patient samples and combined it with fluorescence intensity measurements to generate a fitted curve (Figure S6). The coefficient of determination (R²) reached 0.9959, indicating a strong linear correlation between the G12V (c.35G>T) mutation rate and fluorescence intensity in the patient samples. In addition, we compared the performance of SPEAR with the commonly used qPCR-based detection methods (Figure S7). The qPCR results showed that the amplification curves and ct values of the 1%, 0.1%, and 0.01% mutant samples were indistinguishable from those of the WT samples. The ct values of 100%, 50%, 25%, and 10% mutant samples showed a strong linear correlation with the mutation ratios (logarithmic scale, log). The above analysis indicated that the qPCR-based single nucleotide mutation detection method could quantitatively detect mutant samples ranging from 10% to 100%, but its sensitivity was only 10%. The SPEAR method showed 100 times higher sensitivity for detecting mutations from high background interference compared to the qPCR method. Therefore, the proposed SPEAR method holds high specificity and sensitivity for detecting cancer-related SNPs.

### Clinical sample testing with SPEAR method for practical applications

**Figure 6:**
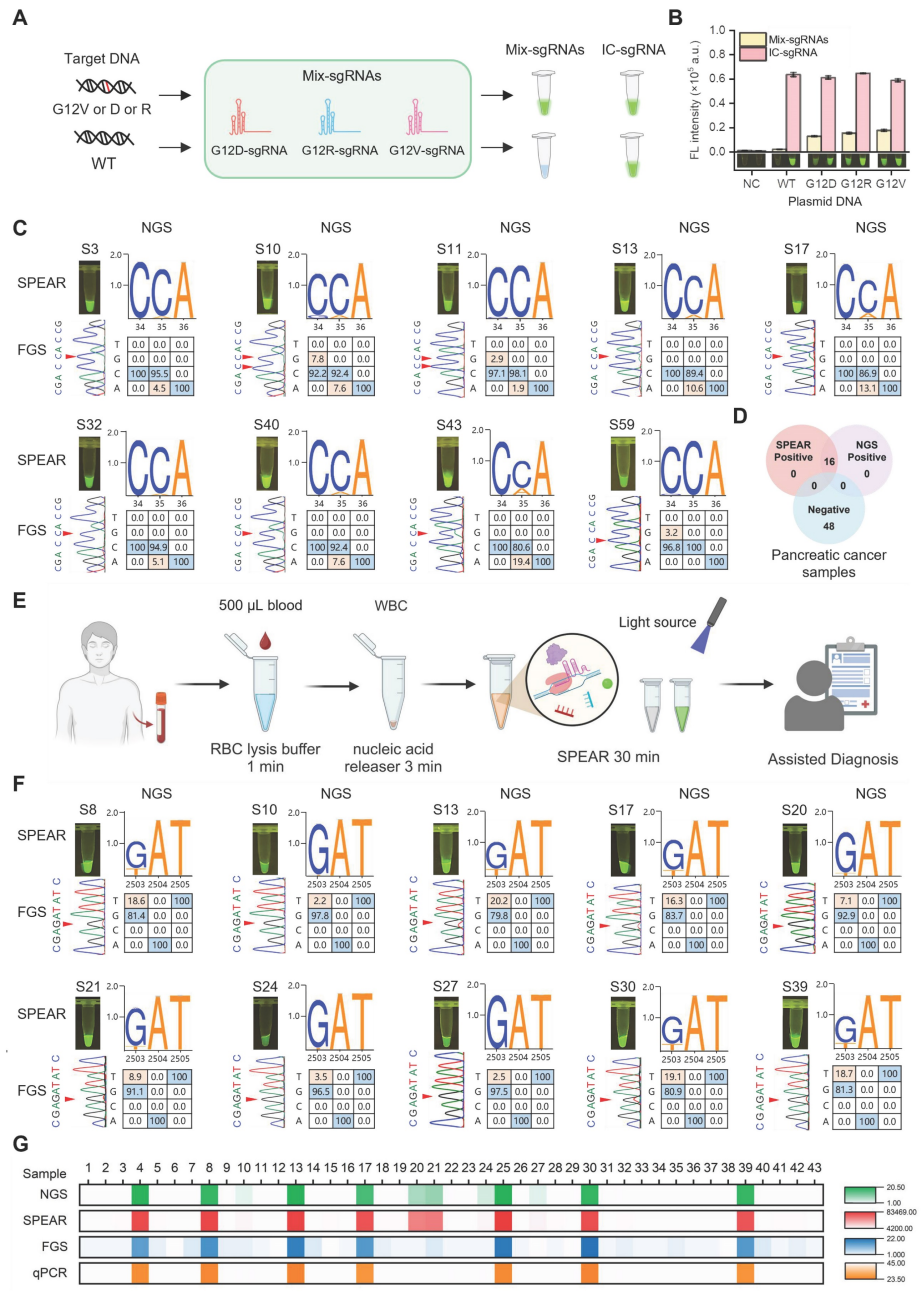
Robust performance of the SPEAR assay for detection of cancer-related SNPs in clinical blood samples. *KRAS* mutations (G12V (c.35G>T)/R (c.34G>C)/D (c.35G>A)) detection with mix-sgRNAs (mix-sgRNAs refers to the mixture of G12V (c.35G>T)/R (c.34G>C)/D (c.35G>A) sgRNAs). (A) Schematic diagram of mix-sgRNAs-guided SPEAR to identify G12V (c.35G>T)/R (c.34G>C)/D (c.35G>A) mutations from WT background. (B) Specificity evaluation of mix-sgRNAs using 1e4 copies of *KRAS* G12V (c.35G>T)/R (c.34G>C)/D (c.35G>A) and WT plasmids. Fluorescence intensity after 30 min of SPEAR reaction was shown. IC, inner control. NC, no template control. Data were the mean ± SD (n = 3). (C) Detection results of representative PC clinical samples using the NGS, FGS, and SPEAR method. The visualization results of SPEAR are displayed on the upper left. The FGS peak diagrams are shown on the lower left, with mutant bases marked by red triangles. The genotypes and mutation rates detected by NGS are shown on the right, with WT and mutated bases marked in blue and orange, respectively. NGS displays the 3' to 5' sequence of the *KRAS* G12 gene, while FGS shows the 3' to 5' sequence of the *KRAS* A11, G12, and G13 regions. (D) Venn diagram showing the results of SPEAR and NGS for detecting KRAS G12V/D/R mutations in clinical pancreatic cancer samples. (E) Schematic diagram of the complete diagnostic process for the *FLT*3 D835 mutations in AML patients (Image created with BioRender.com, with permission). (F) Detection results of representative AML clinical samples using the NGS, FGS, and SPEAR method. The annotation of the figure is the same as (C). NGS displays the sequence of *FLT*3 D835 gene, while FGS shows the sequence of *FLT*3 R834, D835, and I836 genes. (G) Results of NGS, SPEAR, FGS, and qPCR for detecting* FLT*3 D835 mutations in blood samples from 43 AML patients. From top to bottom: mutation rates detected by NGS, fluorescence intensity detected by SPEAR, mutation rates detected by FGS, and ct values detected by qPCR, all visualized using heatmaps.

We challenged the proposed SPEAR method for hotspot mutation detection in pancreatic cancer. The *KRAS* oncogene is known to play a crucial role in the initiation and progression of pancreatic cancer, with G12D (c.35G>A)/V (c.35G>T)/R (c.34G>C) mutations accounting for more than 88.8% of mutation cases 6. In order to target these specific mutant types, we designed three sgRNAs and mixed them together for SPEAR detection (Figure 6A-B). We compared the detection results of SPEAR with FGS, and used NGS as standard reference to evaluate the accuracy of the SPEAR method. Among 64 pancreatic cancer samples, 16 mutation-positive samples detected by NGS were also identified by SPEAR, with no false positives or false negatives, demonstrating 100% sensitivity and specificity. In contrast, FGS detected mutations in just 4 samples, predominantly those with high mutation frequencies, and failed to identify mutations below 10% (Figure S8-S10). In the nine representative samples provided, FGS only identified three with the high mutation rate: S13 (10.6%), S17 (13.1%), S43 (19.4). Notably, the NGS showed that S10, S11 had both G12V (c.35G>T) and G12R (c.34G>C) mutations, and S59 contained 3.2% G12R (c.34G>C) mutation. SPEAR showed positive signals, but none of them were detected by FGS, further confirming the ability of SPEAR system to detect multiple hot spot mutations (Figure 6C-D). These results demonstrate that the SPEAR method exhibits superior sensitivity compared to FGS in the detection of hotspot mutations in clinical pancreatic cancer samples.

To evaluate the versatility of the SPEAR method, we extend it to detect acute myeloid leukemia (AML) resistance gene. *FLT*3 gene mutations D835Y (c.2503G>T)/H (c.2503G>C)/V (c.2504A>T)/F (c.2503G, c.2504A>c.2503T, c.2504T), found in more than one-third of AML cases, may influence the interaction with clinically approved AML inhibitors used in treatment 24. We investigated the specific sgRNAs targeting *FLT*3 D835Y (c.2503G>T)/H (c.2503G>C)/V (c.2504A>T)/F (c.2503G, c.2504A>c.2503T, c.2504T) sites, respectively. Results showed that the 17 nt sgRNA exhibited good specificity at *FLT*3 D835H/V/F sites, but not at the *FLT*3 D835Y (c.2503G>T) site. Therefore, we introduced mismatches into the 17-20 nt sgRNAs to enhance their specificity (Table S1). The results indicated that the 18 nt sgRNA with introduced mismatches exhibited good specificity and high efficiency at the *FLT*3 D835Y (c.2503G>T) site. Thus, we used *FLT*3 D835Y (c.2503G>T) 18 nt sgRNA with artificially introduced mismatch for following assay (Figure S11). To improve the convenience of this method for point-of-care testing, we simplified the whole detection process. 500 µL blood sample was first treated with 1500 µL of red blood cell lysis buffer for 1 min at room temperature. The cell precipitate was then collected by centrifugation at 12,000 rpm for 30 s. Genomic DNA was subsequently released from the cells using 100 µL of nucleic acid releaser at 95 °C for 3 min. Finally, 2 µL of the obtained cell lysate was directly used as the template for the SPEAR assay. The entire process, from blood sampling to result output, could be completed in approximately 30 min (Figure 6E). A mixture of *FLT*3 D835Y (c.2503G>T)/H (c.2503G>C)/V (c.2504A>T)/F (c.2503G, c.2504A>c.2503T, c.2504T) sgRNAs was also utilized to detect clinical samples. We then simultaneously applied SPEAR and FGS to detect 43 AML samples whose *FLT*3 gene mutation status had been analyzed by NGS previously. Among the 43 AML samples, 12 mutation-positive samples were detected by both NGS and SPEAR, while 31 mutation-negative samples were not detected by either method, with no false positives or false negatives, showing 100% sensitivity and specificity (Figure S12-S14). The FGS could only detect 7 clinical samples with a higher mutation rate (>10%) among the NGS-positive cases, but it could not distinguish five positive samples with lower mutation rates (S10, 2.2%; S20, 7.1%; S21, 8.9%; S24, 3.5%; S27, 2.5% *FLT*3 D835Y (c.2503G>T)) (Figure 6F-G). In addition, the commonly used qPCR method was able to detect mutations in only 7 samples with a mutation rate greater than 10%. Therefore, the SPEAR strategy proposed in this paper has higher sensitivity and specificity for clinical sample detection than FGS and qPCR methods, emphasizing the promising potential of SPEAR in detecting cancer-related SNPs.

We further extended the SPEAR method for detecting hotspot mutations in diffuse large B-cell lymphoma (DLBCL). *MYD*88 L265P mutation is the most frequent and most oncogenic form in DLBCL 25. The *MYD*88 L265P 16 nt sgRNA employed in SPEAR enables detection of the *MYD*88 L265P mutation at level as low as 0.1%, meeting clinical requirements (Figure S15). Additionally, all instruments required is a small centrifuge, a mini vortex meter, a pipette and needle, a thermal resistor, and a blue light with a wavelength of 485 nm (Figure S16). The simplified instruments and rapid workflow, along with ultrahigh sensitivity and specificity, makes the SPEAR method a promising tool for point-of-care applications.

## Discussion

Liquid biopsy offers significant advantages over imaging and tissue biopsy by being non-invasive, enabling early cancer detection, and monitoring disease progression and recurrence. In clinical practice, the gene detection technologies based on liquid biopsy currently include NGS, ARMS-PCR, and ddPCR. NGS technology offers high sensitivity and accuracy; however, its long processing time and complex procedures make it challenging for cancer screening applications 7. ARMS-PCR and ddPCR are commonly used methods for detecting SNPs in the laboratory, but they still require large thermal cyclers and specialized operators 9, 11. Additionally, primer and probe design present a significant challenge when performing multiplex detection of the same locus and adjacent loci.

The emergence of CRISPR/Cas systems with base resolution specificity has prompted the development of various nucleic acid detection methods. The integration of amplification techniques such as polymerase chain reaction (PCR), loop-mediated isothermal amplification (LAMP), and recombinase polymerase amplification (RPA) with CRISPR technology has resulted in a complementary win-win situation. On one hand, it enhances the sensitivity of CRISPR-based detection, while on the other, it addresses the specificity limitations of amplification techniques. Unlike the SPEAR system developed in this study, the LAMP system involves a more complicated process for primer design 26. RPA reactions exhibit poor compatibility with the CRISPR system. They rely on the formation of single-stranded structures between primers and templates during integration 27. However, the Cas12a protein has limited single-base resolution when binding to single-stranded nucleic acids, hindering its ability to accurately detect SNPs 28. The RCA reaction has relatively low efficiency and requires longer reaction times 29. In contrast, the SPEAR reaction we proposed can produce observable results within 10 min and achieve single-molecule level detection in approximately 30 min, demonstrating ultra-high sensitivity. Moreover, it is capable of detecting 0.1% mutations in high background concentrations, exhibiting extremely high specificity. The simple primer design and minimal instrument requirements make it a convenient and viable option for cancer screening and prognosis assessment.

In the SPEAR method, we integrated CRISPR-based single base recognition with NEAR amplification. NEAR amplification excels in reaction speed, but its application is significantly constrained by the complexity of primer design and the severity of non-specific amplification. In the SPEAR system, we use a pair of primers to initiate exponential amplification, greatly simplifying primer design. Each primer consists of three components: a stabilization region, a restriction endonuclease site, and a target-binding sequence. To effectively start the reaction, it only requires 17 bases aligned with the template as the target-binding sequence, eliminating the need for further optimization. This makes it suitable for detecting any template sequence, significantly expanding the application scope of the SPEAR method. Moreover, NEAR amplification has encountered significant challenges with non-specific amplification primarily attributed to the simultaneous presence of Bst polymerase, primers, dNTPs, and nicking enzyme. The synergistic action of polymerase and nicking enzyme leads to ab initial DNA synthesis, causing NEAR to significantly amplify non-specific products 30. Using high-resolution polyacrylamide gel electrophoresis to distinguish NEAR products reveals that the molecular weights of some non-specific and specific products are very similar, making the results difficult to differentiate. Even when employing the more precise method of capillary electrophoresis, distinguishing between specific and non-specific products remains challenging 20. Here, we integrated CRISPR with the NEAR reaction, enabling specific detection and even single-base differentiation. To explore the potential for non-specific amplification in the SPEAR system and its implications on false positives or elevated background signals, we conducted SPEAR reactions with extended reaction times. Remarkably, even when the reaction time was extended to one hour, no background fluorescence was generated. This result strongly indicates the exceptional specificity of the SPEAR system.

Our study showed that SPEAR is an exceptionally sensitive and specific method for mutation detection. It offers several advantages: no risk of aerosol contamination, a constant temperature reaction without the need for specialized instruments, faster reaction time and easier operation, thereby enhancing its utility for immediate cancer screening and precision medicine applications. While it also has some limitations. Specifically, the system's universality is influenced by the presence of the PAM motif and the positioning of SNPs within the crRNA region. It is expected that ongoing advancements in the CRISPR toolbox will address these challenges in the future.

## Conclusion

In summary, we developed a novel method called SPEAR for detecting cancer-related SNPs, which could also be extended to other mutation types. SPEAR exhibited unparalleled features as follows: 1) It provides a quite simple nucleic acid detection system with simplified primer design and basic components, by reaction at isothermal condition and the whole detection process could be accomplished in only 30 min; 2) Its detection sensitivity is nearly at the single molecule level, capable of detecting targets at a ratio as low as 0.1% under strong background interference; 3) It enables discrimination of single-base mutations and allows for multiple detection in one tube without cross-interference. It can be easily extended to the analysis of other cancer-related mutations for cancer screening and prognostic assessment. 4) It is quite convenient which only require a thermal block and a blue light, with bulk instruments and professional operators abandoned. Therefore, the proposed SPEAR method possessed exceptional sensitivity, specificity, speed, and convenience, showing great potential for point-of-care cancer screening and precision medicine applications.

## Supplementary Material

Supplementary materials and methods, figures and tables.

## Abbreviations

SPEAR: Specific Point mutation Evaluation via CRISPR-Cas Assisted Recognition
CRISPR: clustered regularly interspaced short palindromic repeats
Cas: CRISPR-associated
sgRNA: single guide RNA
dsDNA: double-stranded DNA
ssDNA: single-stranded DNA
NEAR: nicking enzyme amplification reaction
SNPs: single nucleotide polymorphisms
FGS: first-generation sequencing
NGS: next-generation sequencing
PC: pancreatic cancer
AML: acute myeloid leukemia
DLBCL: diffuse large b-cell lymphoma
ARMS-PCR: amplification refractory mutation system-polymerase chain reaction
ddPCR: droplet digital PCR
LOD: limit of detection
cfDNA: cell free DNA
