# Supplementary material for: SPEAR: CRISPR-mediated ultrasensitive, specific and rapid one-pot detection strategy for cancer-related SNPs
Source: Theranostics. 2025 Feb 18;15(8):3275–88. doi: 10.7150/thno.107488 (PMC11905141; doi:10.7150/thno.107488)
Supplement: Supplementary file 1 — Supplementary materials and methods, figures and tables. [file thnov15p3275s1.pdf]

Supplementary Materials

SPEAR: CRISPR-mediated ultrasensitive, specific and rapid one-pot detection strategy for cancer-related SNPs

Linlin Bai[1†], Yanan Pang[2†], Ting Wang[3†], Shengzhou Wang[1], Kaiming Guo[1], Tian Xuan,[1] Ziqin Zhang[4], Dianwei Liu[1], Feng Qian[1], Yan Zheng[1], Gang Jin[5, *], Rui Wang[1, 6*]

[1]Center for Medical Research and Innovation, Shanghai Pudong Hospital, Human Phenome Institute, State Key Laboratory of Genetic Engineering, School of Life Sciences, School of Pharmacy, Fudan University, Shanghai, 200438, China

[2]Department of Gastroenterology, Shanghai Institute of Pancreatic Diseases, Changhai Hospital; National Key Laboratory of Immunity and Inflammation, Naval Medical University, Shanghai, 200433, China

[3]Department of Hematology, Peking University Shenzhen Hospital, Shenzhen Peking University-The Hong Kong University of Science and Technology Medical Center, Shenzhen, 518036, China

[4]College of Biological Science and Engineering, Fuzhou University, Fuzhou, 350108, China

[5]Department of Hepatobiliary Pancreatic Surgery, Changhai Hospital, Naval Medical University, Shanghai 200433, China

[6]International Human Phenome Institutes, Shanghai, 200433, China

† These authors contributed equally.

*Corresponding authors

R. Wang E-mail address: wangr@fudan.edu.cn

J. Gang E-mail address: jingang@smmu.edu.cn

Materials and Methods

The sgRNA preparation

Single guide RNA (sgRNA) was synthesized and purified with T7 Transcription Kit. Firstly, the template was amplified via PCR assay. Then, 2 μL of T7 polymerase, 10 μL of NTP buffer and 4 μL of the PCR product were mixed, and DEPC water was added to make up a 30 μL transcription system. This mixture was then incubated at 37 °C for 24 h to transcribe the DNA template into RNA. Next, 50 μL reaction system including DNase I 5 μL ,10 × DNase I buffer 5 μL, RNase inhibitor 20 units was heated at 37 °C for 30 min to ensure complete digestion of the DNA template. Then, the obtained RNA products were purified using HiPure RNA Pure Micro Kit, following the operational instructions. Finally, the RNA was eluted into RNase-free water and preserved at −80 °C refrigerator.

Cell culture and nucleic acid extraction

Pancreatic cancer cell line PaTu8988 and human fetal hepatocyte line 293T were cultured in petri dishes. The culture medium was 5 mL DMEM containing 100 U/mL penicillin-streptomycin and 10% FBS. Cells were cultured at 37 °C humidified atmosphere containing 5% CO_2. Total DNA of cell samples was extracted using the TIANGEN Genomic DNA Kit (input volume 200 μL, elution volume 50 μL).

Preparation of mutant mock DNA samples

Plasmid Mutant Mock DNA Samples: Mutant and wild-type (WT) templates were mixed at various ratios, with the *FLT*3 D835Y mutation comprising 100%, 50%, 25%, 10%, 1%, 0.1%, and 0.01% (i.e., 100 copies of the D835Y template in a total of 1,000,000 templates) of the total template pool. A total of 1×10^6 copies of the mixed plasmid DNA was used as input for the SPEAR assay.

Cell Line Genomic DNA Mutation-Mocked Samples: Genomic DNA extracted from the PaTu8988 cell line (containing the G12V mutation) was mixed with wild-type genomic DNA from

blood cells to generate mutant concentrations ranging from 1% to 0.01%. A total of 80 ng of the genomic DNA was used as input for the SPEAR assay.

The cfDNA Mutation-Mocked Samples: ctDNA containing 19.4% G12V mutations from plasma samples of pancreatic cancer patients was mixed with wild-type ctDNA from healthy individuals to prepare ctDNA mutation-doped samples with mutation concentrations ranging from 19.4% to 0.01%. A total of 80 ng of genomic DNA was used as input for the SPEAR assay.

Polyacrylamide Gel Electrophoresis Analysis

After the SPEAR reaction, 10 μL product was mixed with 10 × loading buffer. Electrophoresis was carried out on 20% polyacrylamide gel with 5 bp DNA Ladder (Thermo Fisher Scientific, MD, USA). Electrophoresis was conducted at 110 V constant voltage for 2 h. After electrophoresis, the gel was immersed in 0.1 M NaCl solution with 3 × GelRed nucleic acid dye (Biomed, Beijing, China) for 30 min and subsequently imaged using the ChemiDoc™ XRS+ Imager (Bio-Rad, California, USA).

First-generation sequencing (FGS) and next-generation sequencing (NGS)

For FGS, the extracted DNA was amplified by PCR. The amplification system contained *TaKaRa Taq* HS (5 U/μL) 0.25 μL, 10 × PCR Buffer (Mg^{2+} plus) 5 μL, dNTP Mixture (each 2.5 mM) 5 μL, Template 3 μL, primer F/R 400 nM (Specific primer sequences were shown in Table S4), and DEPC H_2O to make up the final volume to 50 μL. The PCR procedure was 95 °C 5 min, (95 °C 20s, 55 °C 30s, 72 °C 30s) × 40 cycles, and 72 °C 5 min. 20 μl PCR products were sent for FGS by Sangon Biotech (Shanghai, China). The mutation rate was obtained through quantitative analysis of the FGS sequencing map using the website https://moriaritylab.shinyapps.io/editr_v10/.

For NGS, Firstly, the extracted DNA was amplified by PCR with primers containing barcodes. The amplification system contained 2 × PCR buffer for KOD Fx 25 μL, 2 mM dNTPs 10 μL, 10 μM Primer F/R 1.5 μL (Specific primer sequences were shown in Table S5), Template 5 μL, KOD Fx (1.0 U/μL) 1 μL, and DEPC H_2O to make up the final volume to 50 μL. First round PCR procedure was 94 °C 2 min, (98 °C 10 s, 55 °C 30 s, 68 °C 30 s) × 25 cycles, and 68 °C 7 min. In

the second round of PCR, different barcode primers were used to amplify the KTAS G12 and *FLT*3-D835 region of samples. The amplification system and procedure were as follows, 2 × PCR buffer for KOD Fx 25 μL, 2mM dNTPs 10 μL, 10 μM Primer F/R 1.5 μL (Specific primer sequences were shown in Table S5), 6 μL Amplicons of the first round PCR, KOD Fx (1.0 U/μL) 1 μL, and DEPC H_2O to 50 μL. The second round PCR procedure was 94 °C 2 min, (98 °C 10 s， 57 °C 30 s， 68 °C 30 s) × 15 cycles, 68 °C 7 min. The PCR products were purified by TIANgel Midi Purification Kit, and sent to Mingma Technologies (Shanghai， China) for NGS.

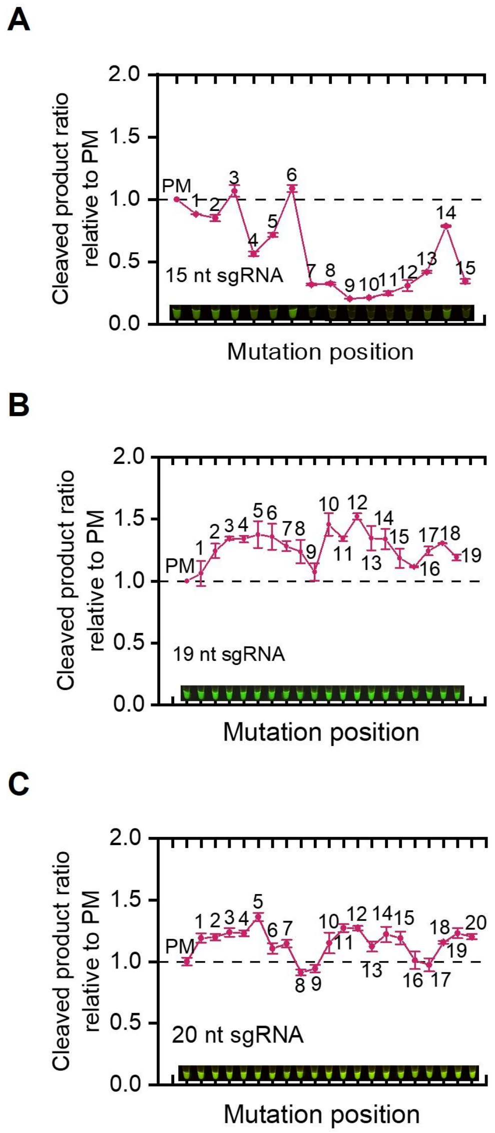

Figure S1. Specificity evaluation of AapCas12b cleavage with different length of sgRNAs. (A) 15 nt sgRNA, (B) 19 nt sgRNA, (C) 20 nt sgRNA. Data were the mean ± SD (n = 3).

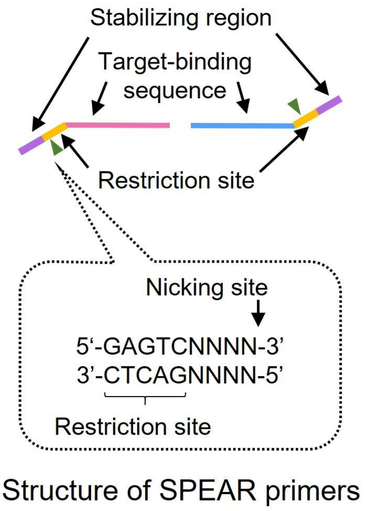

Figure S2. Structure of primers in SPEAR assay. Each primer consists of three parts: a stabilizing region, a restriction site, and a target-binding sequence. The function of the stabilizing region is to stabilize the binding of the restriction enzyme with the primer and template during the amplification process. The restriction site is identified by nicking enzyme. The target-binding sequence is the part where the primer complementary pairing with the target. The restriction enzyme cleavage site is located between the 4th and 5th positions near the restriction site (GAGTC).

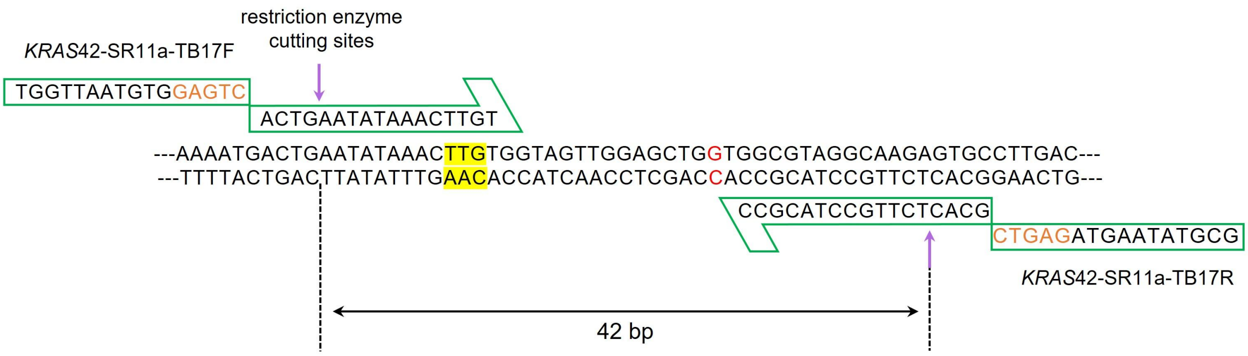

Figure S3. Schematic diagram illustrating primer binding and target amplification of the *KRAS* G12V gene. The yellow-filled region represents the PAM sequence, while the red font indicates the mutation site.

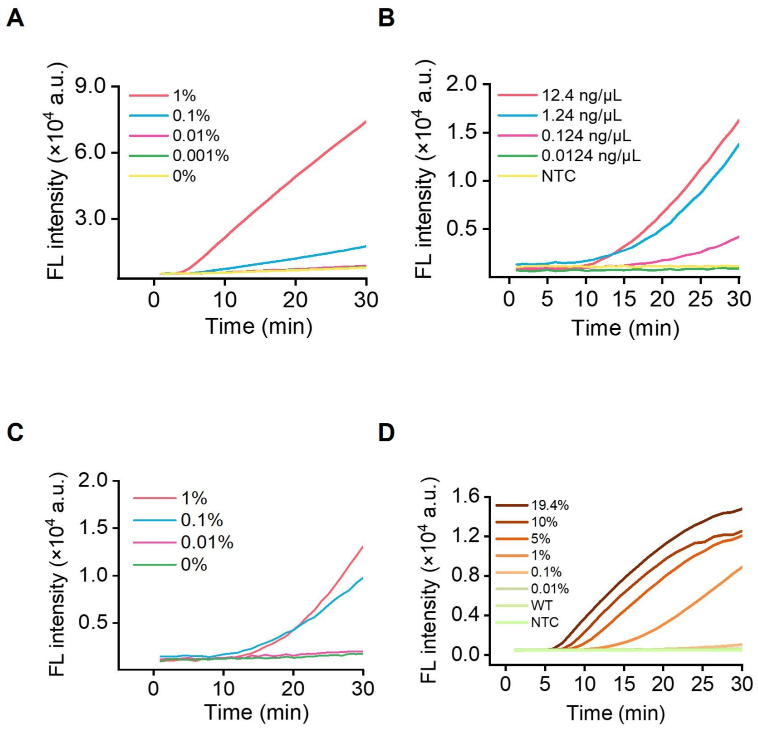

Figure S4. Sensitivity evaluation of SPEAR method. (A) Sensitivity of SPEAR method in detecting 1e6 copies of plasmid templates with gradient *KRAS* G12V mutation rates. The real-time fluorescence was presented. (B) Detection sensitivity of genomic DNA of PaTu8988 cell line using SPEAR method. The real-time fluorescence was presented. (C) Sensitivity of mutation rate detection with genomic DNA as template, a mixture of PaTu8988 and 293T genomic DNA, totaling 80 ng and containing gradient *KRAS* G12V mutation rates, was used as templates for SPEAR detection. The real-time fluorescence was presented. (D) Sensitivity of mutation rate detection with cfDNA as template. A mixture of cfDNA extracted from pancreatic cancer patients and healthy individuals, totaling 80 ng and containing gradient *KRAS* G12V mutation rates, was used as templates for SPEAR detection. NTC: no template control. Data were the mean ± SD (n = 3).

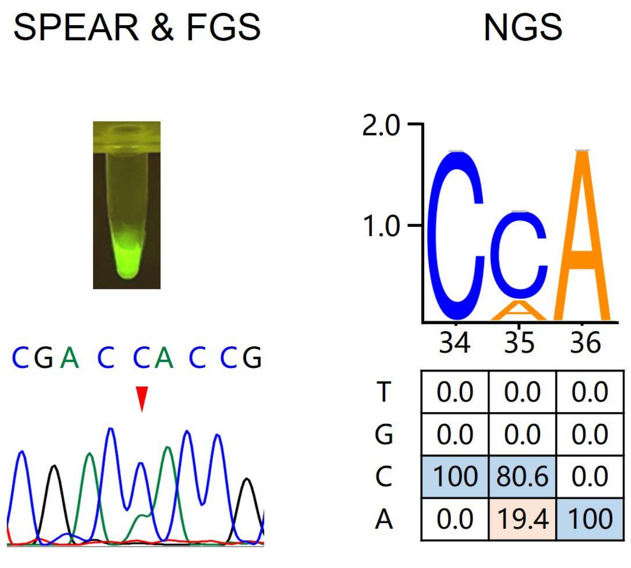

,

Figure S5. Detection results of a PC clinical sample using the NGS, FGS, and SPEAR method. The visualization result of the SPEAR method is displayed on the upper left. The FGS peak diagrams is shown on the lower left, with mutant base marked by red triangle. The genotype and mutation rate detected by NGS are shown on the right, WT and mutated bases were marked with blue and orange, respectively.

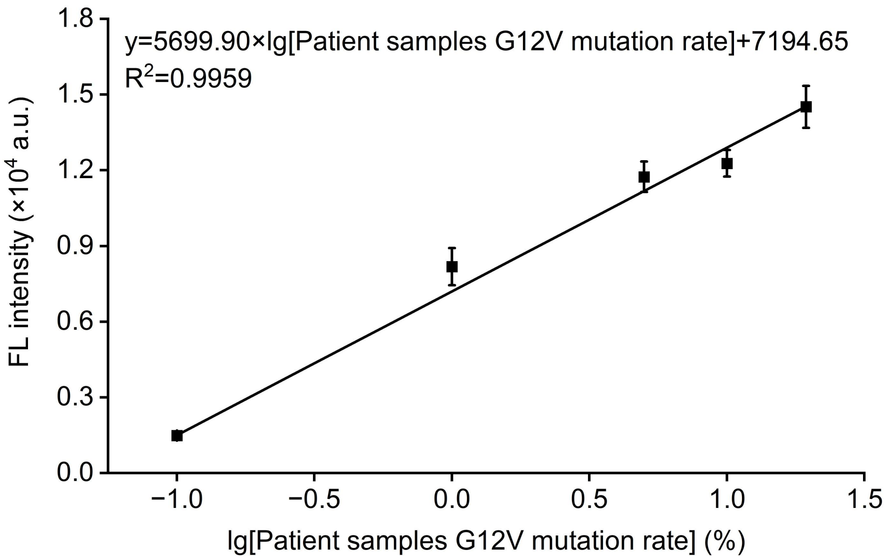

Figure S6. The fitted curve between patient sample G12V mutation rate (lg) and fluorescence intensity.

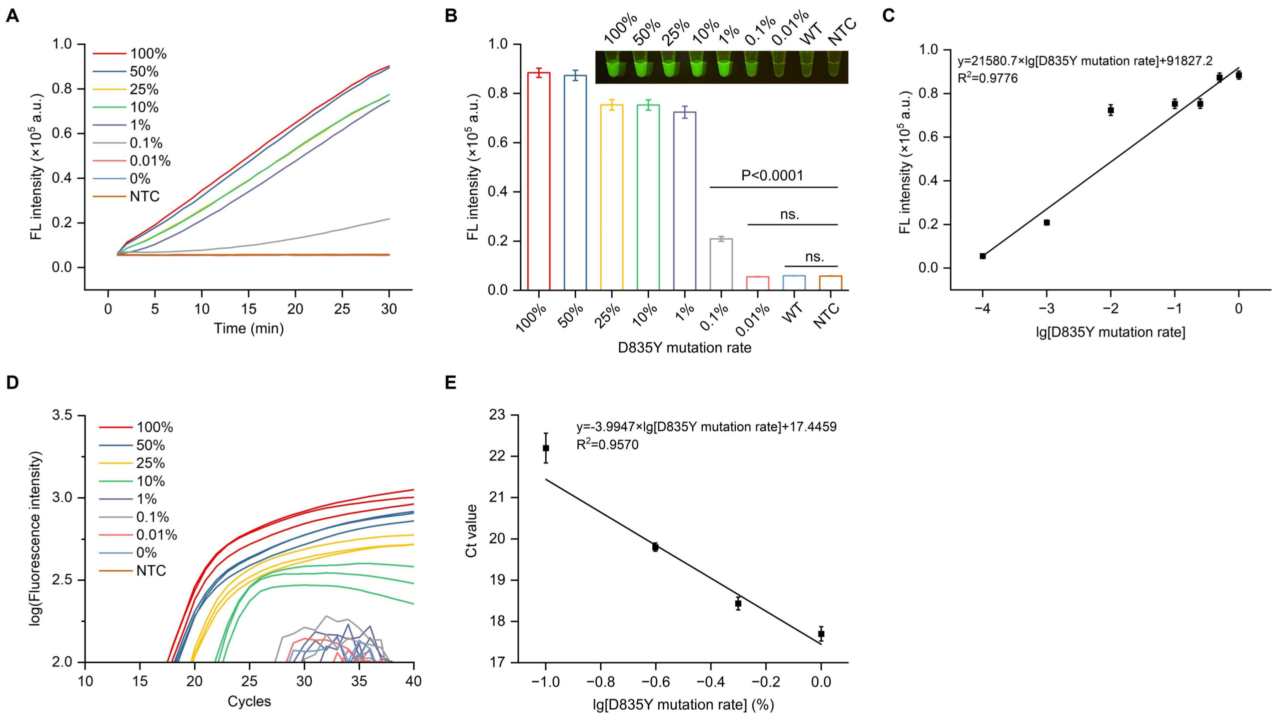

Figure S7. SPEAR and qPCR were used to detect the D835Y mutation rates. (A) Sensitivity of SPEAR method in detecting 1e6 copies of plasmid templates with gradient *FLT* 3 D835Y mutation rates. The real-time fluorescence was presented. (B) The 30 min endpoint fluorescence values and visualization results. (C) The fitted curve between 0.01%, 0.1%, 1%, 10%, 25%, 50%, 100% D835Y mutation rates (lg) and the corresponding fluorescence intensity. (D) The qPCR amplification plot of 1e6 copies of plasmid templates with gradient D835Y mutation rates. (E) The fitted curve between 10%, 25%, 50%, 100% *FLT*3 D835Y mutation rates (lg) and the corresponding ct values. Data are the mean ± SD (n = 3).

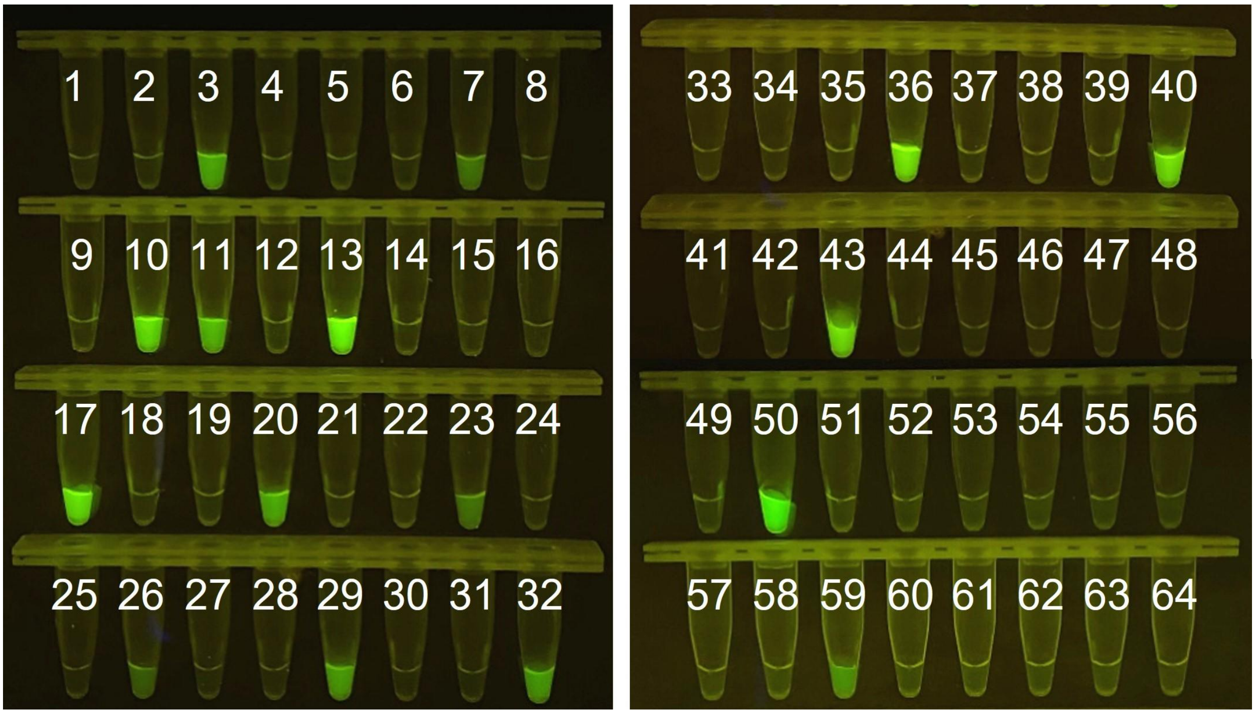

Figure S8. SPEAR results of 64 PC patient samples observed by naked eyes under 485 nm blue light.

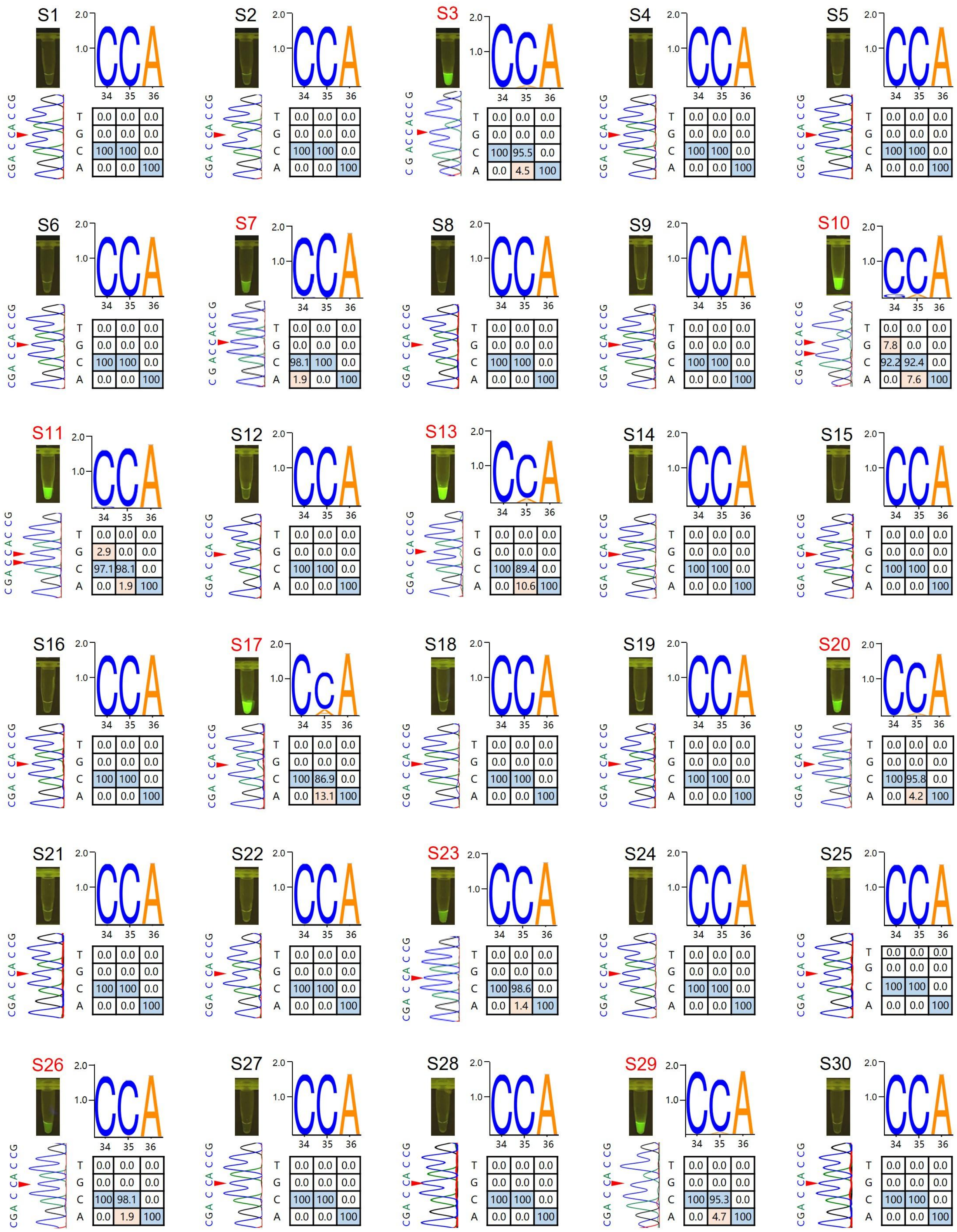
S1 T 0.0 0.0 0.0 G 0.0 0.0 0.0 C 100 100 0.0 A 0.0 0.0 100
S2 T 0.0 0.0 0.0 G 0.0 0.0 0.0 C 100 100 0.0 A 0.0 0.0 100
S3 T 0.0 0.0 0.0 G 0.0 0.0 0.0 C 100 95.5 0.0 A 0.0 4.5 100
S4 T 0.0 0.0 0.0 G 0.0 0.0 0.0 C 100 100 0.0 A 0.0 0.0 100
S5 T 0.0 0.0 0.0 G 0.0 0.0 0.0 C 100 100 0.0 A 0.0 0.0 100
S6 T 0.0 0.0 0.0 G 0.0 0.0 0.0 C 100 100 0.0 A 0.0 0.0 100
S7 T 0.0 0.0 0.0 G 0.0 0.0 0.0 C 98.1 100 0.0 A 1.9 0.0 100
S8 T 0.0 0.0 0.0 G 0.0 0.0 0.0 C 100 100 0.0 A 0.0 0.0 100
S9 T 0.0 0.0 0.0 G 0.0 0.0 0.0 C 100 100 0.0 A 0.0 0.0 100
S10 T 0.0 0.0 0.0 G 7.8 0.0 0.0 C 92.2 92.4 0.0 A 0.0 7.6 100
S11 T 0.0 0.0 0.0 G 2.9 0.0 0.0 C 97.1 98.1 0.0 A 0.0 1.9 100
S12 T 0.0 0.0 0.0 G 0.0 0.0 0.0 C 100 100 0.0 A 0.0 0.0 100
S13 T 0.0 0.0 0.0 G 0.0 0.0 0.0 C 100 89.4 0.0 A 0.0 10.6 100
S14 T 0.0 0.0 0.0 G 0.0 0.0 0.0 C 100 100 0.0 A 0.0 0.0 100
S15 T 0.0 0.0 0.0 G 0.0 0.0 0.0 C 100 100 0.0 A 0.0 0.0 100
S16 T 0.0 0.0 0.0 G 0.0 0.0 0.0 C 100 100 0.0 A 0.0 0.0 100
S17 T 0.0 0.0 0.0 G 0.0 0.0 0.0 C 100 86.9 0.0 A 0.0 13.1 100
S18 T 0.0 0.0 0.0 G 0.0 0.0 0.0 C 100 100 0.0 A 0.0 0.0 100
S19 T 0.0 0.0 0.0 G 0.0 0.0 0.0 C 100 100 0.0 A 0.0 0.0 100
S20 T 0.0 0.0 0.0 G 0.0 0.0 0.0 C 100 95.8 0.0 A 0.0 4.2 100
S21 T 0.0 0.0 0.0 G 0.0 0.0 0.0 C 100 100 0.0 A 0.0 0.0 100
S22 T 0.0 0.0 0.0 G 0.0 0.0 0.0 C 100 100 0.0 A 0.0 0.0 100
S23 T 0.0 0.0 0.0 G 0.0 0.0 0.0 C 100 98.6 0.0 A 0.0 1.4 100
S24 T 0.0 0.0 0.0 G 0.0 0.0 0.0 C 100 100 0.0 A 0.0 0.0 100
S25 T 0.0 0.0 0.0 G 0.0 0.0 0.0 C 100 100 0.0 A 0.0 0.0 100
S26 T 0.0 0.0 0.0 G 0.0 0.0 0.0 C 100 98.1 0.0 A 0.0 1.9 100
S27 T 0.0 0.0 0.0 G 0.0 0.0 0.0 C 100 100 0.0 A 0.0 0.0 100
S28 T 0.0 0.0 0.0 G 0.0 0.0 0.0 C 100 100 0.0 A 0.0 0.0 100
S29 T 0.0 0.0 0.0 G 0.0 0.0 0.0 C 100 95.3 0.0 A 0.0 4.7 100
S30 T 0.0 0.0 0.0 G 0.0 0.0 0.0 C 100 100 0.0 A 0.0 0.0 100

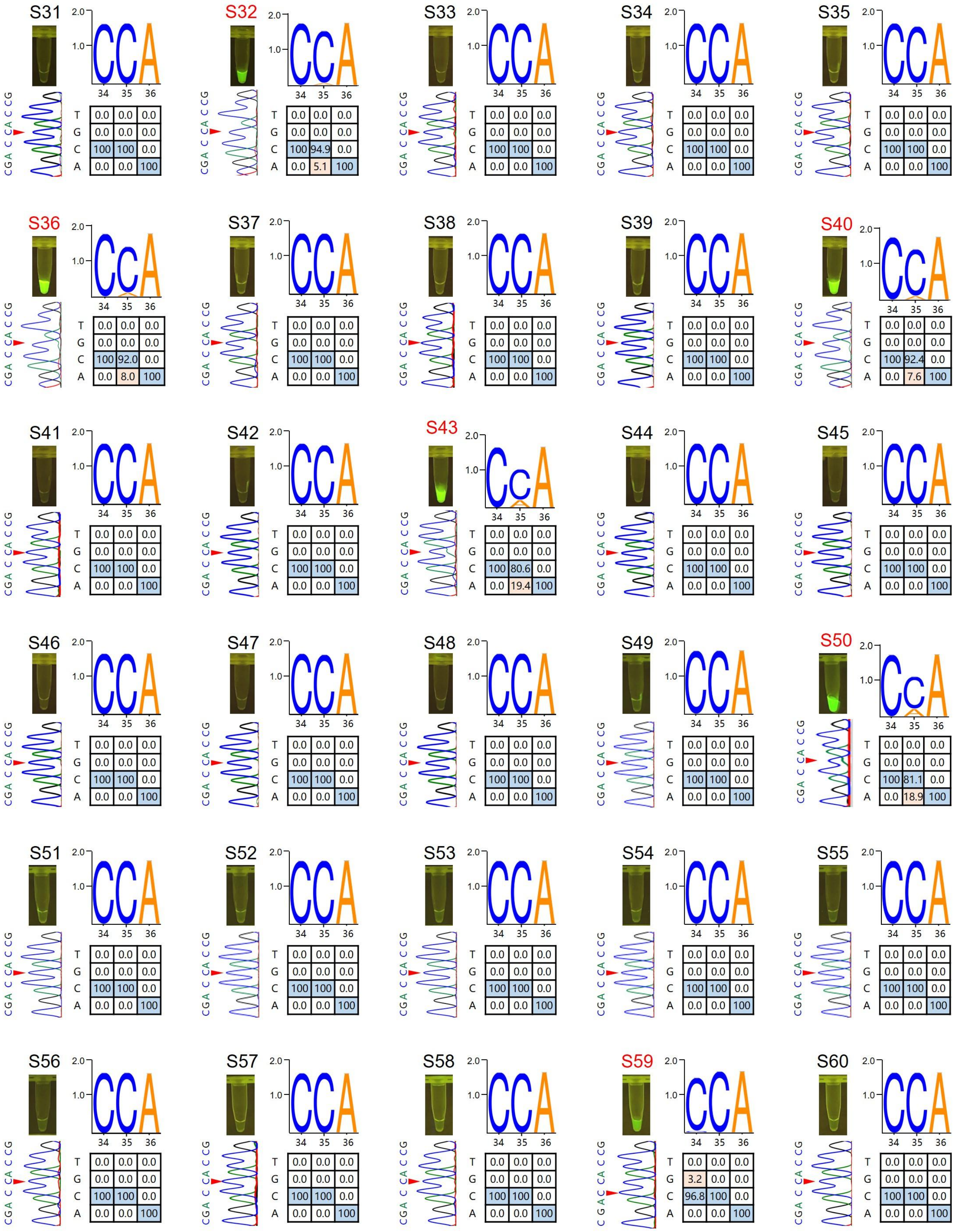

S31
T 0.0 0.0 0.0
G 0.0 0.0 0.0
C 100 100 0.0
A 0.0 0.0 100
S32
T 0.0 0.0 0.0
G 0.0 0.0 0.0
C 100 94.9 0.0
A 0.0 5.1 100
S33
T 0.0 0.0 0.0
G 0.0 0.0 0.0
C 100 100 0.0
A 0.0 0.0 100
S34
T 0.0 0.0 0.0
G 0.0 0.0 0.0
C 100 100 0.0
A 0.0 0.0 100
S35
T 0.0 0.0 0.0
G 0.0 0.0 0.0
C 100 100 0.0
A 0.0 0.0 100
S36
T 0.0 0.0 0.0
G 0.0 0.0 0.0
C 100 92.0 0.0
A 0.0 8.0 100
S37
T 0.0 0.0 0.0
G 0.0 0.0 0.0
C 100 100 0.0
A 0.0 0.0 100
S38
T 0.0 0.0 0.0
G 0.0 0.0 0.0
C 100 100 0.0
A 0.0 0.0 100
S39
T 0.0 0.0 0.0
G 0.0 0.0 0.0
C 100 100 0.0
A 0.0 0.0 100
S40
T 0.0 0.0 0.0
G 0.0 0.0 0.0
C 100 92.4 0.0
A 0.0 7.6 100
S41
T 0.0 0.0 0.0
G 0.0 0.0 0.0
C 100 100 0.0
A 0.0 0.0 100
S42
T 0.0 0.0 0.0
G 0.0 0.0 0.0
C 100 100 0.0
A 0.0 0.0 100
S43
T 0.0 0.0 0.0
G 0.0 0.0 0.0
C 100 80.6 0.0
A 0.0 19.4 100
S44
T 0.0 0.0 0.0
G 0.0 0.0 0.0
C 100 100 0.0
A 0.0 0.0 100
S45
T 0.0 0.0 0.0
G 0.0 0.0 0.0
C 100 100 0.0
A 0.0 0.0 100
S46
T 0.0 0.0 0.0
G 0.0 0.0 0.0
C 100 100 0.0
A 0.0 0.0 100
S47
T 0.0 0.0 0.0
G 0.0 0.0 0.0
C 100 100 0.0
A 0.0 0.0 100
S48
T 0.0 0.0 0.0
G 0.0 0.0 0.0
C 100 100 0.0
A 0.0 0.0 100
S49
T 0.0 0.0 0.0
G 0.0 0.0 0.0
C 100 100 0.0
A 0.0 0.0 100
S50
T 0.0 0.0 0.0
G 0.0 0.0 0.0
C 100 81.1 0.0
A 0.0 18.9 100
S51
T 0.0 0.0 0.0
G 0.0 0.0 0.0
C 100 100 0.0
A 0.0 0.0 100
S52
T 0.0 0.0 0.0
G 0.0 0.0 0.0
C 100 100 0.0
A 0.0 0.0 100
S53
T 0.0 0.0 0.0
G 0.0 0.0 0.0
C 100 100 0.0
A 0.0 0.0 100
S54
T 0.0 0.0 0.0
G 0.0 0.0 0.0
C 100 100 0.0
A 0.0 0.0 100
S55
T 0.0 0.0 0.0
G 0.0 0.0 0.0
C 100 100 0.0
A 0.0 0.0 100
S56
T 0.0 0.0 0.0
G 0.0 0.0 0.0
C 100 100 0.0
A 0.0 0.0 100
S57
T 0.0 0.0 0.0
G 0.0 0.0 0.0
C 100 100 0.0
A 0.0 0.0 100
S58
T 0.0 0.0 0.0
G 0.0 0.0 0.0
C 100 100 0.0
A 0.0 0.0 100
S59
T 0.0 0.0 0.0
G 3.2 0.0 0.0
C 96.8 100 0.0
A 0.0 0.0 100
S60
T 0.0 0.0 0.0
G 0.0 0.0 0.0
C 100 100 0.0
A 0.0 0.0 100

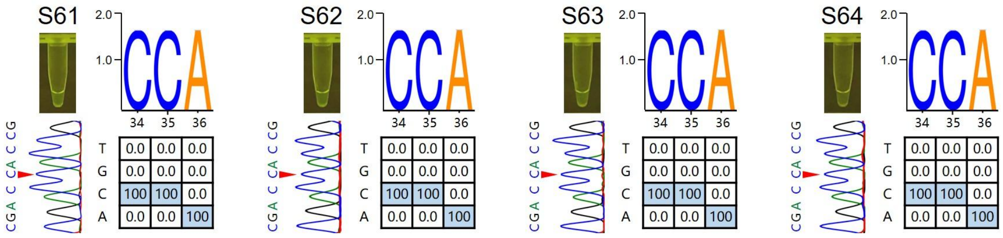

Figure S9. SPEAR, FGS and NGS results of 64 PC patient blood samples. The visualization results of the SPEAR method are displayed on the upper left. The FGS peak diagrams are shown on the lower left, with mutant bases marked by red triangles. The genotypes and mutation rates detected by NGS are shown on the right, WT and mutated bases are marked with blue and orange, respectively. NGS displays the 3’-5’ sequence of the *KRAS* G12 gene, while FGS shows the 3’-5’ sequence of the *KRAS* A11, G12, and G13 genes.

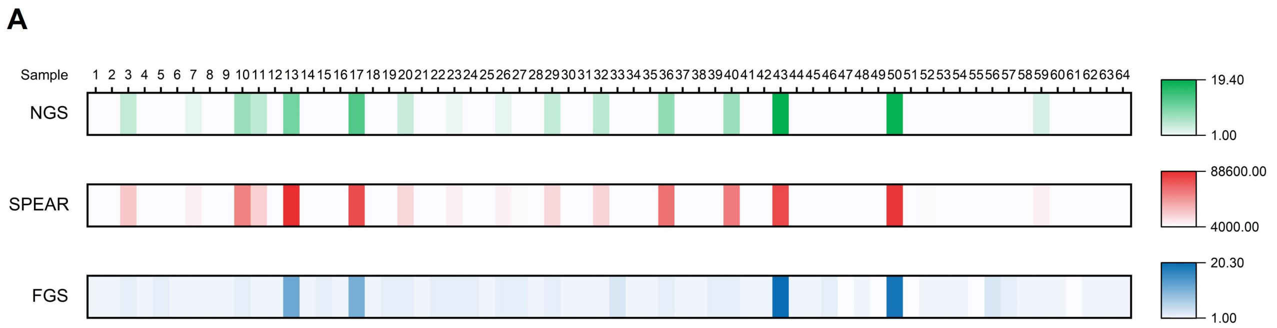

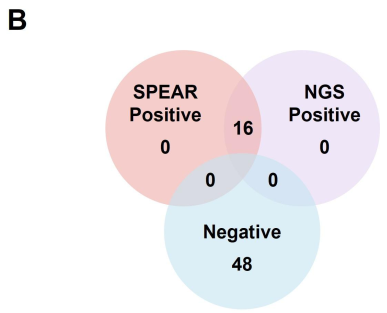

C

Methods		NGS		Total
		Positive	Negative	
**SPEAR**	Positive	16	0	16
	Negative	0	48	48
	Total	16	48	64

Sensitivity: 100%; Specificity: 100%; Accuracy: 100%; PPV: 100%; NPV: 100%;

Figure S10. (A) Results of NGS, SPEAR, and FGS for detecting mutations in blood samples from 64 patients with pancreatic cancer. From top to bottom: heat map analysis of mutation rates detected by NGS, heat map analysis of fluorescence intensity detected by SPEAR, and heat map analysis of mutation rates detected by FGS (FGS sequencing map quantitatively analyzed using the website https://moriaritylab.shinyapps.io/editr_v10/). (B) Venn diagram showing the results of SPEAR and NGS for detecting KRAS G12V/D/R mutations in clinical pancreatic cancer samples. (C) The negative predictive value (NPV), positive predictive value (PPV), sensitivity, and specificity of SPEAR for detecting KRAS G12V/D/R mutations in clinical pancreatic cancer samples.

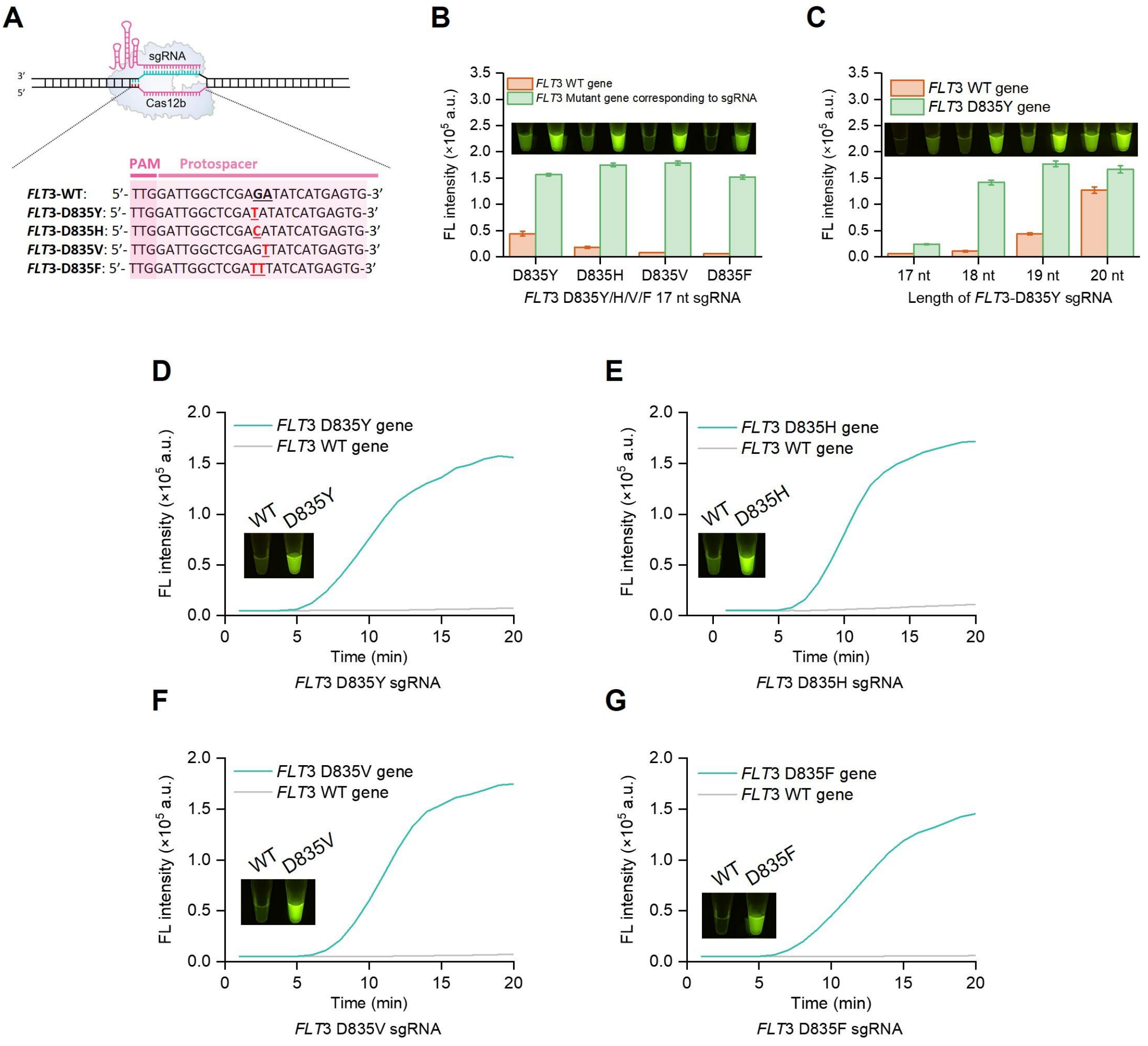

Figure S11. The application of SPEAR in identifying AML *FLT*3 D835Y/H/V/F mutations. (A) Sequence information of *FLT*3 D835Y/H/V/F. (B) Exploring the optimal sgRNAs targeting *FLT*3 mutations D835Y/H/V/F individually. The 17 nt sgRNAs were designed to target the D835Y/H/V/F mutations. The 30 min endpoint fluorescence values and visualization results are presented. (C) Specificity of 17, 18, 19, 20 nt *FLT*3-D835Y sgRNA with artificially introduced mutation. The 30 min endpoint fluorescence values and visualization results are provided. d-g the optimal sgRNAs were used to detect *FLT*3 mutations D835Y (D), D835H (E), D835F (F), and D835V (G) in the SPEAR reaction. The real-time fluorescence and visualization results are presented. The sequence information of the optimal sgRNAs is shown in Table S3. Data were the mean ± SD (n = 3).

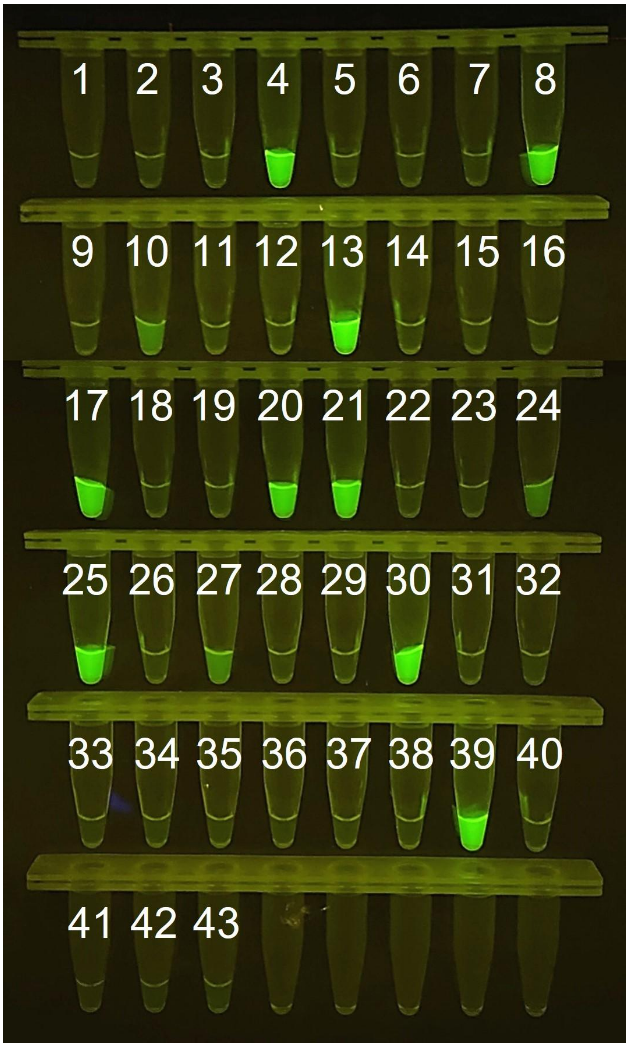

Figure S12. SPEAR results of 43 AML patient samples observed by naked eyes under 485 nm blue light.

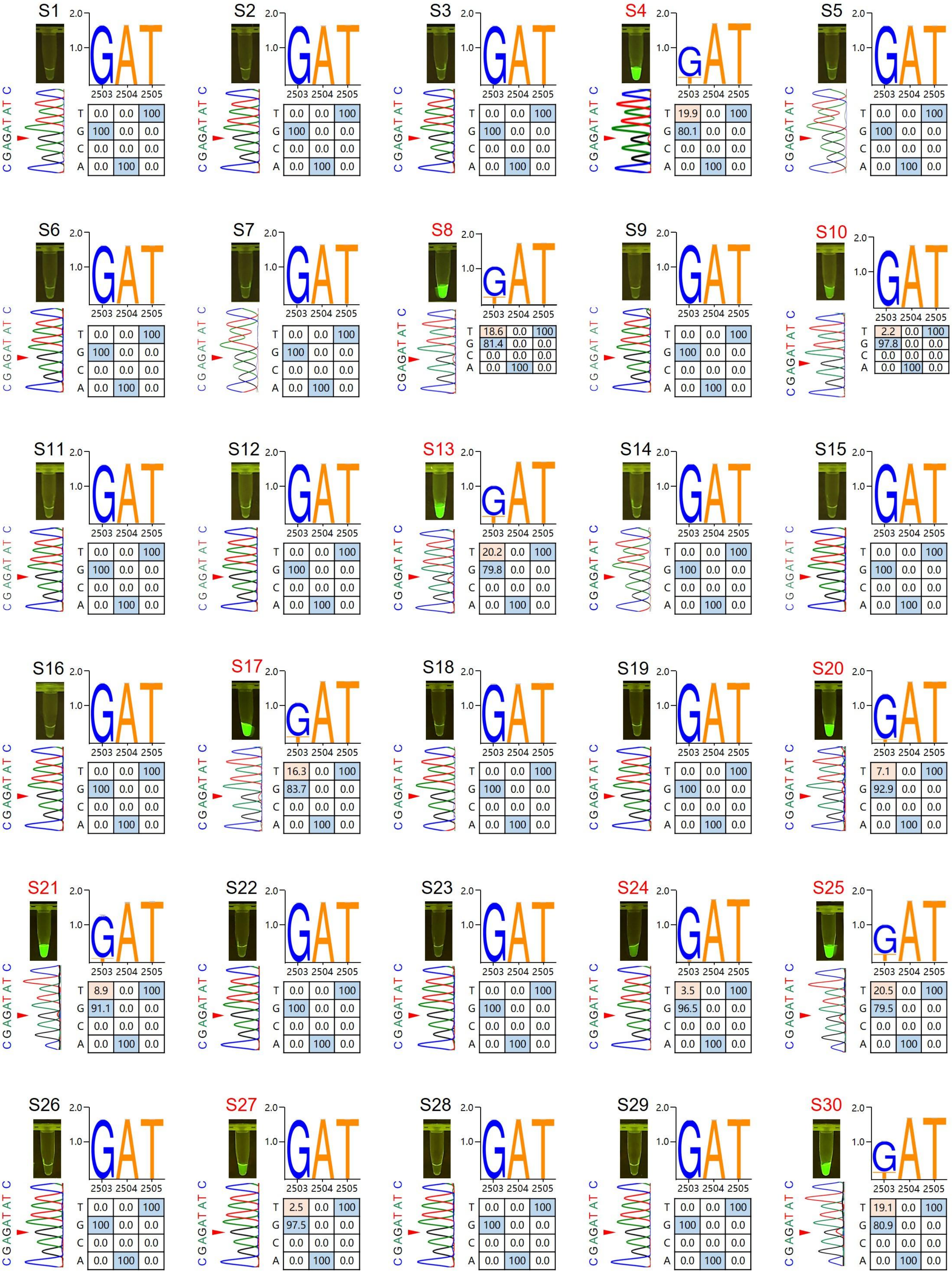
S1
S2
S3
S4
S5
S6
S7
S8
S9
S10
S11
S12
S13
S14
S15
S16
S17
S18
S19
S20
S21
S22
S23
S24
S25
S26
S27
S28
S29
S30
2503 2504 2505
CGAGATATC

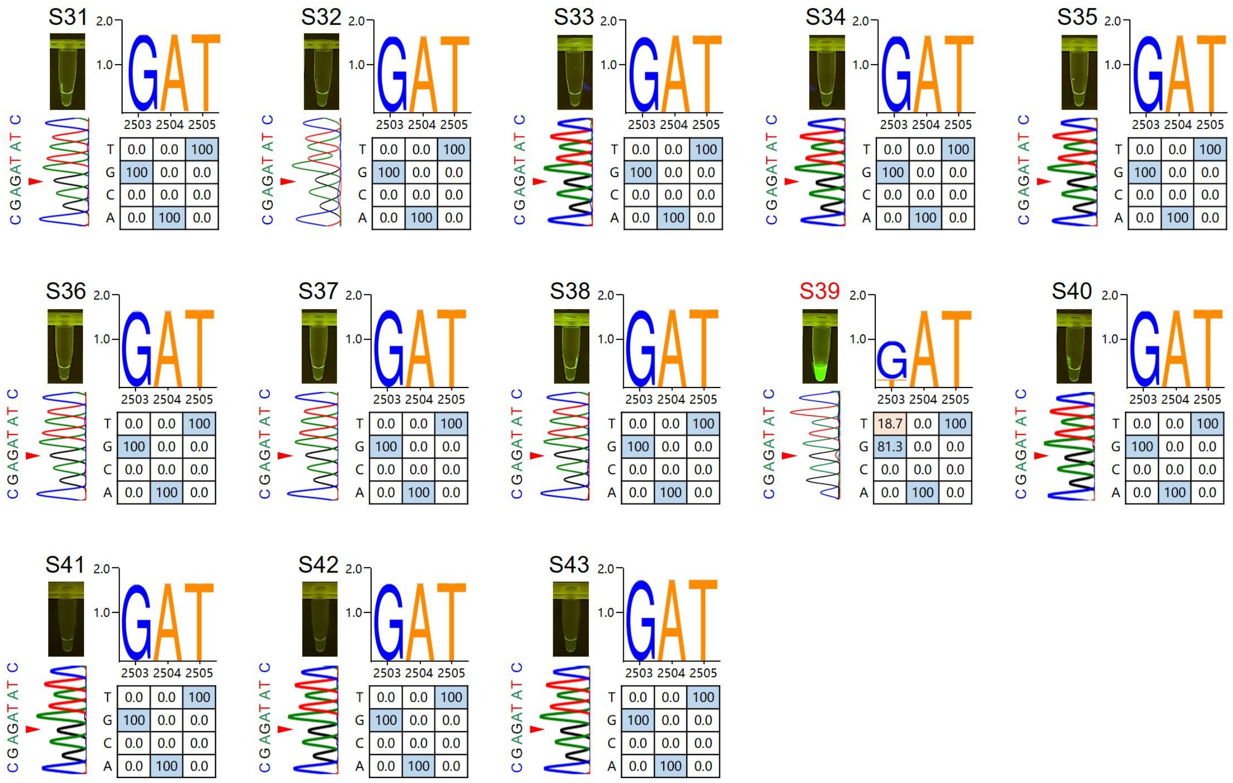

Figure S13. SPEAR, FGS and NGS results of 43 AML patient blood samples. The visualization results of the SPEAR method are displayed on the upper left. The FGS peak diagrams are shown on the lower left, with mutant bases marked by red triangles. The genotypes and mutation rates detected by NGS are shown on the right, WT and mutated bases were marked with blue and orange, respectively. NGS displays the sequence of *FLT*3 D835 gene, while FGS shows the sequence of *FLT*3 R834, D835, and I836 genes.

A

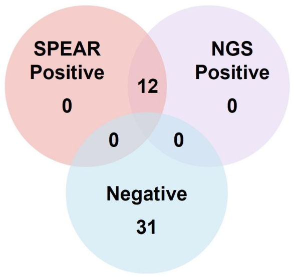

B

Methods		NGS		Total
		Positive	Negative	
SPEAR	Positive	12	0	12
	Negative	0	31	31
	Total	12	31	43

Sensitivity: 100%; Specificity: 100%; Accuracy: 100%; PPV: 100%; NPV: 100%;

Figure S14. (A) Venn diagram showing the results of SPEAR and NGS for *FLT*3 D835Y/V/F/H mutations detection in clinical AML samples. (B) NPV, PPV, sensitivity, and specificity of SPEAR for detecting the *FLT*3 D835Y/V/F/H mutation in clinical AML samples.

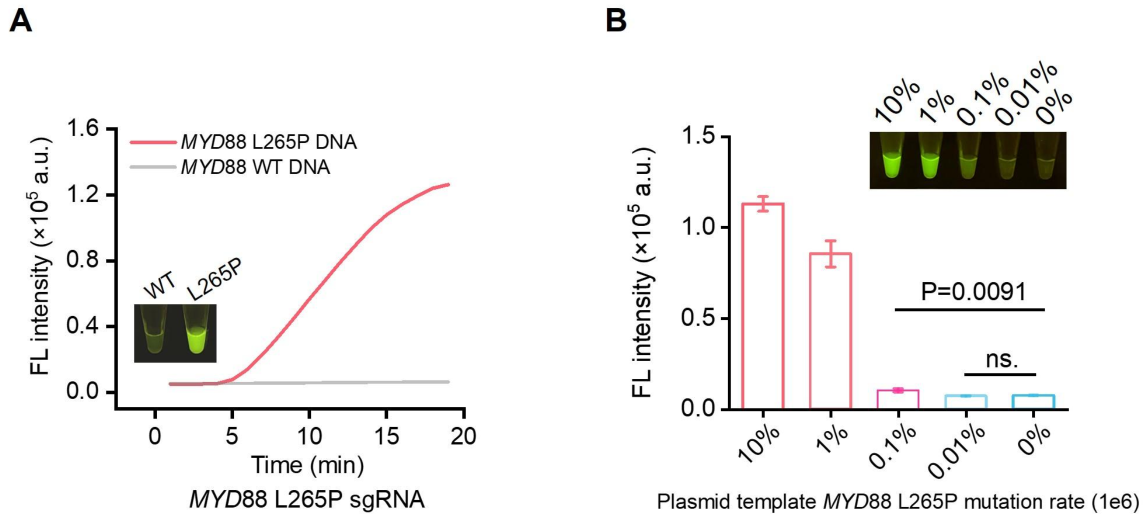

Figure S15. The application of SPEAR in identifying DLBCL *MYD*88 L265P mutations. (A) The specific sgRNA was used to detect the *MYD*88 L265P gene in the SPEAR reaction. The real-time fluorescence and visualization results are presented. (B) Sensitivity of SPEAR method in detecting 1e6 copies of plasmid templates with gradient *MYD*88 L265P mutation rates. The 30 min endpoint fluorescence values and visualization results are demonstrated. Data were the mean ± SD (n = 3).

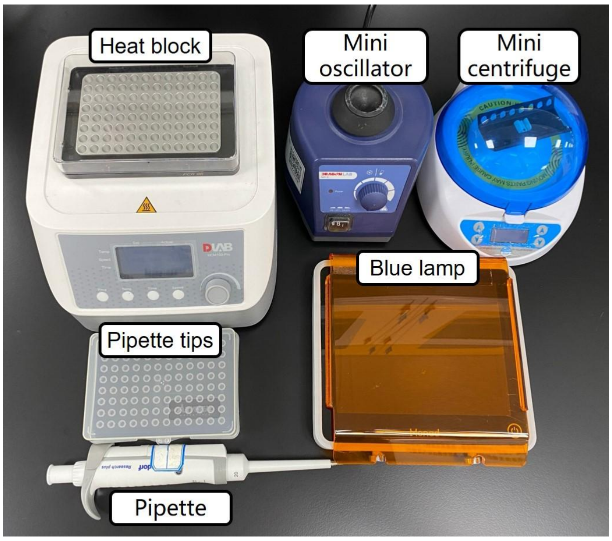

Figure S16. Equipment needed in SPEAR assay.

Table S1.

Sequence information for *in vitro* sgRNA transcription

Name	*Sequence (5′ -3′)
sgRNA-T7-universal	TAATACGACTCACTATAGGG
AapCas12b-sgRNA	TAATACGACTCACTATAGGGGTCTAGAGGACAGAATTTTTCAA CGGGTGTGCCAATGGCCACTTTCCAGGTGGCAAAGCCCGTTGA GCTTCTCAAATCTGAGAAGTGGCACGTAGCAGCAAGATTAGCA GAAGCT
KRAS-12b-WT-20gR	GCCACCAGCTCCAACTACCA-GTGCCACTTCTCAGATTTGA
KRAS-12b-WT-19gR	CCACCAGCTCCAACTACCA-GTGCCACTTCTCAGATTTGA
KRAS-12b-WT-18gR	CACCAGCTCCAACTACCA-GTGCCACTTCTCAGATTTGA
KRAS-12b-WT-17gR	ACCAGCTCCAACTACCA-GTGCCACTTCTCAGATTTGA
KRAS-12b-WT-16gR	CCAGCTCCAACTACCA-GTGCCACTTCTCAGATTTGA
KRAS-12b-WT-15gR	CAGCTCCAACTACCA-GTGCCACTTCTCAGATTTGA
KRAS-12b-G12V-18gR	CAACAGCTCCAACTACCA-GTGCCACTTCTCAGATTTGA
KRAS-12b-G12V-17gR	AACAGCTCCAACTACCA-GTGCCACTTCTCAGATTTGA
KRAS-12b-G12V-16gR	ACAGCTCCAACTACCA-GTGCCACTTCTCAGATTTGA
KRAS-12b-G12D-18gR	CATCAGCTCCAACTACCA-GTGCCACTTCTCAGATTTGA
KRAS-12b-G12D-17gR	ATCAGCTCCAACTACCA-GTGCCACTTCTCAGATTTGA
KRAS-12b-G12D-16gR	TCAGCTCCAACTACCA-GTGCCACTTCTCAGATTTGA
KRAS-12b-G12R-18gR	CACGAGCTCCAACTACCA-GTGCCACTTCTCAGATTTGA
KRAS-12b-G12R-17gR	ACGAGCTCCAACTACCA-GTGCCACTTCTCAGATTTGA
KRAS-12b-G12R-16gR	CGAGCTCCAACTACCA-GTGCCACTTCTCAGATTTGA
KRAS-12b-G12R-15gR	GAGCTCCAACTACCA-GTGCCACTTCTCAGATTTGA
FLT3-12b-D835Y-17gR	GATATATCGAGCCAATC-GTGCCACTTCTCAGATTTGA
FLT3-12b-D835H-17gR	GATATGTCGAGCCAATC-GTGCCACTTCTCAGATTTGA
FLT3-12b-D835V-17gR	GATAACTCGAGCCAATC-GTGCCACTTCTCAGATTTGA
FLT3-12b-D835F-17gR	GATAAATCGAGCCAATC-GTGCCACTTCTCAGATTTGA
FLT3-12b-D835Y-17MR	GATGTATCGAGCCAATC-GTGCCACTTCTCAGATTTGA
FLT3-12b-D835Y-18MR	TGATGTATCGAGCCAATC-GTGCCACTTCTCAGATTTGA
FLT3-12b-D835Y-19MR	ATGATGTATCGAGCCAATC-GTGCCACTTCTCAGATTTGA
FLT3-12b-D835Y-20MR	CATGATGTATCGAGCCAATC-GTGCCACTTCTCAGATTTG
MYD88-12b-L265P-17gR	CCGATCCCCATCAAGTA-GTGCCACTTCTCAGATTTGA
MYD88-12b-L265P-16gR	CGATCCCCATCAAGTA-GTGCCACTTCTCAGATTTGA

* The T7 promoter sequence is in green. The sequence before the dash is a template for the Cas12b sgRNA spacer sequence. The mutation sites are marked by red color different from spacer sequences, and the artificially introduced mutation sites are marked by yellow fill. The DNA template of the optimal sgRNA corresponding to each target is marked with an underline.

Table S2.

Oligonucleotide Sequence of dsDNA templates with SNP

Name	*Sequence (5′ -3′)
KRAS Target-wtF	ATGTATGACCAGTTGAGATTGTGGTAGTTGGAGCTGGTGGCTGATGAAGACGACTCTGA
KRAS Target-wtR	TCAGAGTCGTCTTCATCAGCCACCAGCTCCAACTACCACAATCTCAACTGGTCATACAT
KRAS Target-mm1F	ATGTATGACCAGTTGAGATTGAGGTAGTTGGAGCTGGTGGCTGATGAAGACGACTCTGA
KRAS Target-mm1R	TCAGAGTCGTCTTCATCAGCCACCAGCTCCAACTACCTCAATCTCAACTGGTCATACAT
KRAS Target-mm2F	ATGTATGACCAGTTGAGATTGTCGTAGTTGGAGCTGGTGGCTGATGAAGACGACTCTGA
KRAS Target-mm2R	TCAGAGTCGTCTTCATCAGCCACCAGCTCCAACTACGACAATCTCAACTGGTCATACAT
KRAS Target-mm3F	ATGTATGACCAGTTGAGATTGTGCTAGTTGGAGCTGGTGGCTGATGAAGACGACTCTGA
KRAS Target-mm3R	TCAGAGTCGTCTTCATCAGCCACCAGCTCCAACTAGCACAATCTCAACTGGTCATACAT
KRAS Target-mm4F	ATGTATGACCAGTTGAGATTGTGGAAGTTGGAGCTGGTGGCTGATGAAGACGACTCTGA
KRAS Target-mm4R	TCAGAGTCGTCTTCATCAGCCACCAGCTCCAACTTCCACAATCTCAACTGGTCATACAT
KRAS Target-mm5F	ATGTATGACCAGTTGAGATTGTGGTTGTTGGAGCTGGTGGCTGATGAAGACGACTCTGA
KRAS Target-mm5R	TCAGAGTCGTCTTCATCAGCCACCAGCTCCAACAACCACAATCTCAACTGGTCATACAT
KRAS Target-mm6F	ATGTATGACCAGTTGAGATTGTGGTACTTGGAGCTGGTGGCTGATGAAGACGACTCTGA
KRAS Target-mm6R	TCAGAGTCGTCTTCATCAGCCACCAGCTCCAAGTACCACAATCTCAACTGGTCATACAT
KRAS Target-mm7F	ATGTATGACCAGTTGAGATTGTGGTAGATGGAGCTGGTGGCTGATGAAGACGACTCTGA
KRAS Target-mm7R	TCAGAGTCGTCTTCATCAGCCACCAGCTCCATCTACCACAATCTCAACTGGTCATACAT
KRAS Target-mm8F	ATGTATGACCAGTTGAGATTGTGGTAGTAGGAGCTGGTGGCTGATGAAGACGACTCTGA
KRAS Target-mm8R	TCAGAGTCGTCTTCATCAGCCACCAGCTCCTACTACCACAATCTCAACTGGTCATACAT
KRAS Target-mm9F	ATGTATGACCAGTTGAGATTGTGGTAGTTCGAGCTGGTGGCTGATGAAGACGACTCTGA
KRAS Target-mm9R	TCAGAGTCGTCTTCATCAGCCACCAGCTCGAACTACCACAATCTCAACTGGTCATACAT
KRAS Target-mm10F	ATGTATGACCAGTTGAGATTGTGGTAGTTGCAGCTGGTGGCTGATGAAGACGACTCTGA
KRAS Target-mm10R	TCAGAGTCGTCTTCATCAGCCACCAGCTGCAACTACCACAATCTCAACTGGTCATACAT
KRAS Target-mm11F	ATGTATGACCAGTTGAGATTGTGGTAGTTGGTGCTGGTGGCTGATGAAGACGACTCTGA
KRAS Target-mm11R	TCAGAGTCGTCTTCATCAGCCACCAGCACCAACTACCACAATCTCAACTGGTCATACAT
KRAS Target-mm12F	ATGTATGACCAGTTGAGATTGTGGTAGTTGGACCTGGTGGCTGATGAAGACGACTCTGA
KRAS Target-mm12R	TCAGAGTCGTCTTCATCAGCCACCAGGTCCAACTACCACAATCTCAACTGGTCATACAT
KRAS Target-mm13F	ATGTATGACCAGTTGAGATTGTGGTAGTTGGAGGTGGTGGCTGATGAAGACGACTCTGA
KRAS Target-mm13R	TCAGAGTCGTCTTCATCAGCCACCACCTCCAACTACCACAATCTCAACTGGTCATACAT
KRAS Target-mm14F	ATGTATGACCAGTTGAGATTGTGGTAGTTGGAGCAGGTGGCTGATGAAGACGACTCTGA
KRAS Target-mm14R	TCAGAGTCGTCTTCATCAGCCACCTGCTCCAACTACCACAATCTCAACTGGTCATACAT
KRAS Target-mm15F	ATGTATGACCAGTTGAGATTGTGGTAGTTGGAGCTCGTGGCTGATGAAGACGACTCTGA
KRAS Target-mm15R	TCAGAGTCGTCTTCATCAGCCACGAGCTCCAACTACCACAATCTCAACTGGTCATACAT
KRAS Target-mm16FA	ATGTATGACCAGTTGAGATTGTGGTAGTTGGAGCTGATGGCTGATGAAGACGACTCTGA
KRAS Target-mm16RA	TCAGAGTCGTCTTCATCAGCCATCAGCTCCAACTACCACAATCTCAACTGGTCATACAT
KRAS Target-mm16FT	ATGTATGACCAGTTGAGATTGTGGTAGTTGGAGCTGTTGGCTGATGAAGACGACTCTGA
KRAS Target-mm16RT	TCAGAGTCGTCTTCATCAGCCAACAGCTCCAACTACCACAATCTCAACTGGTCATACAT
KRAS Target-mm17F	ATGTATGACCAGTTGAGATTGTGGTAGTTGGAGCTGGAGGCTGATGAAGACGACTCTGA

KRAS Target-mm17R	TCAGAGTCGTCTTCATCAGCCTCCAGCTCCAACTACCACAATCTCAACTGGTCATACAT
KRAS Target-mm18F	ATGTATGACCAGTTGAGATTGTGGTAGTTGGAGCTGGTCGCTGATGAAGACGACTCTGA
KRAS Target-mm18R	TCAGAGTCGTCTTCATCAGCGACCAGCTCCAACTACCACAATCTCAACTGGTCATACAT
KRAS Target-mm19F	ATGTATGACCAGTTGAGATTGTGGTAGTTGGAGCTGGTGCCTGATGAAGA CGACTCTGA
KRAS Target-mm19R	TCAGAGTCGTCTTCATCAGGCACCAGCTCCAACTACCACAATCTCAACTGGTCATACAT
KRAS Target-mm20F	ATGTATGACCAGTTGAGATTGTGGTAGTTGGAGCTGGTGGGTGATGAAGACGACTCTGA
KRAS Target-mm20R	TCAGAGTCGTCTTCATCACCCACCAGCTCCAACTACCACAATCTCAACTGGTCATACAT

* PAM sites are highlighted with yellow background, sgRNA recognition sequences are underlined, and mismatch sites are highlighted in red font. Template F and template R are complementary pairs and annealed to form a double-stranded template.

Table S3.

Primer sequences used in SPEAR

Purpose	Name	*Sequence (5′-3′)
Optimization of the length of target-binding sequence	*KRAS*50-SR11-TB11F	TGGTTAATGTGGAGTCAAAATGACTGA
	*KRAS*50-SR11-TB11R	GCGTATAAGTAGAGTCAGGCACTCTTG
	*KRAS*50-SR11-TB13F	TGGTTAATGTGGAGTCAAAATGACTGAAT
	*KRAS*50-SR11-TB13R	GCGTATAAGTAGAGTCAGGCACTCTTGCC
	*KRAS*50-SR11-TB15F	TGGTTAATGTGGAGTCAAAATGACTGAATAT
	*KRAS*50-SR11-TB15R	GCGTATAAGTAGAGTCAGGCACTCTTGCCTA
	*KRAS*50-SR11-TB17F	TGGTTAATGTGGAGTCAAAATGACTGAATATAA
	*KRAS*50-SR11-TB17R	GCGTATAAGTAGAGTCAGGCACTCTTGCCTACG
	*KRAS*50-SR11-TB19F	TGGTTAATGTGGAGTCAAAATGACTGAATATAAAC
	*KRAS*50-SR11-TB19R	GCGTATAAGTAGAGTCAGGCACTCTTGCCTACGCC
Optimization of the length of stabilizing region	*KRAS*50-SR7-TB17F	TAATGTGGAGTCAAAATGACTGAATATAA
	*KRAS*50-SR7-TB17R	ATAAGTAGAGTCAGGCACTCTTGCCTACG
	*KRAS*50-SR9-TB17F	GTTAATGTGGAGTCAAAATGACTGAATATAA
	*KRAS*50-SR9-TB17R	GTATAAGTAGAGTCAGGCACTCTTGCCTACG
	*KRAS*50-SR11-TB17F	TGGTTAATGTGGAGTCAAAATGACTGAATATAA
	*KRAS*50-SR11-TB17R	GCGTATAAGTAGAGTCAGGCACTCTTGCCTACG
	*KRAS*50-SR13-TB17F	TGTGGTTAATGTGGAGTCAAAATGACTGAATATAA
	*KRAS*50-SR13-TB17R	TCGCGTATAAGTAGAGTCAGGCACTCTTGCCTACG
	*KRAS*50-SR15-TB17F	AGTGTGGTTAATGTGGAGTCAAAATGACTGAATATAA
	*KRAS*50-SR15-TB17R	ACTCGCGTATAAGTAGAGTCAGGCACTCTTGCCTACG
	*KRAS*50-SR17-TB17F	ACAGTGTGGTTAATGTGGAGTCAAAATGACTGAATATAA
	*KRAS*50-SR17-TB17R	GTACTCGCGTATAAGTAGAGTCAGGCACTCTTGCCTACG
Optimization of the length of target DNA	*KRAS*38-SR11a-TB17F	TGGTTAATGTGGAGTCTATAAACTTGTGGTAGT
	*KRAS*38-SR11a-TB17R	GCGTATAAGTAGAGTCAGGCACTCTTGCCTACG
	*KRAS*42-SR11a-TB17F	TGGTTAATGTGGAGTCACTGAATATAAACTTGT
	*KRAS*42-SR11a-TB17R	GCGTATAAGTAGAGTCGCACTCTTGCCTACGCC
	*KRAS*45-SR11a-TB17F	TGGTTAATGTGGAGTCCTGAATATAAACTTGTG
	*KRAS*45-SR11a-TB17R	GCGTATAAGTAGAGTCCAAGGCACTCTTGCCTA
	*KRAS*53-SR11a-TB17F	TGGTTAATGTGGAGTCAATGACTGAATATAAAC
	*KRAS*53-SR11a-TB17R	GCGTATAAGTAGAGTCCGTCAAGGCACTCTTGC
Optimization of the stabilizing region sequence features	*KRAS*42-SR11a-TB17F	TGGTTAATGTGGAGTCACTGAATATAAACTTGT
	*KRAS*42-SR11a-TB17R	GCGTATAAGTAGAGTCGCACTCTTGCCTACGCC
	*KRAS*42-SR11b-TB17F	GTGTGCTTCTAGAGTCACTGAATATAAACTTG
	*KRAS*42-SR11b-TB17R	GTGTGCTTCTAGAGTCGCACTCTTGCCTACGCC

	*KRAS*42-SR11c-TB17F	ATGGGTAGGTTGAGTCACTGAATATAAACTTGT
	*KRAS*42-SR11c-TB17R	TGTATTGTATTGAGTCGCACTCTTGCCTACGCC
	*KRAS*42-SR11d-TB17F	GGCCCTGTCTTGAGTCACTGAATATAAACTTGT
	*KRAS*42-SR11d-TB17R	GGTCCTGTTCTGAGTCGCACTCTTGCCTACGCC
*FLT*3 primer	*FLT*3/42-SR11a-TB17F	TGGTTAATGTGGAGTCAAGATATGTGACTTTGG
	*FLT*3/42-SR11a-TB17F	GCGTATAAGTAGAGTCTAGTTGGAATCACTCAT
*MYD*88 primer	*MYD*88/40-SR11a-TB17F	TGGTTAATGTGGAGTCGTGCCCATCAGAAGCGA
	*MYD*88/40-SR11a-TB17R	GCGTATAAGTAGAGTCTCTTCATTGCCTTGTAC

* Taking *KRAS*50-SR11-TB11F as an example to introduce the naming of primers: *KRAS* refers to the target gene, 50 indicates the length of the amplification product in the SPEAR system, SR denotes Stabilizing Region, followed by 11 indicating the length of the Stabilizing Region, TB stands for Target-Binding Sequence, followed by 11 indicating the length of the Target-Binding Sequence, and F denotes the forward primer. The naming of other primers follows the same meaning as this example. Different color markings indicate the optimal primer pairs for this gene.

Table S4.

Primer sequences for First generation sequencing

Target	Name	*Sequence (5′-3′)
KRAS gene	*KRAS*-FGS-F	ATGACTGAATATAAACTTGT
	KRAS-NGS-R	CTCTATTGTTGGATCATATT
*FLT*3 gene	*FLT*3-FGS-F	TCGTGTGTTCACAGAGACCT
	*FLT*3-FGS-R	GGCATTGCCCCTGACAACATAGT
*FLT*3 D835Y TaqMan probe	D835Y-TaqMan	FAM-CTCGATATATCATGAGTG-MGB

* D835Y mutated bases were colored in red.

Table S5.

Primer sequences for Next-generation sequencing

Name	*Sequence (5′-3′)
KRAS-NGS-F	ACACTCTTTCCCTACACGACGCTCTTCCGATCTNNNNATGACTGAATATAAACTTGT
KRAS-NGS-R	ACTGGAGTTCAGACGTGTGCTCTTCCGATCTNNNNCTCTATTGTTGGATCATATT
*FLT*3-NGS-F	ACACTCTTTCCCTACACGACGCTCTTCCGATCTNNNNTCGTGTGTTCACAGAGACCT
*FLT*3-NGS-R	ACTGGAGTTCAGACGTGTGCTCTTCCGATCTNNNNGGCATTGCCCCTGACAACATAGT
P5-index1-F	AATGATACGGCGACCACCGAGATCTACACTGAACCTTACACTCTTTCCCTACACGAC
P5-index2-F	AATGATACGGCGACCACCGAGATCTACACTGCTAAGTACACTCTTTCCCTACACGAC
P5-index3-F	AATGATACGGCGACCACCGAGATCTACACTAAGACACACACTCTTTCCCTACACGAC
P5-index4-F	AATGATACGGCGACCACCGAGATCTACACTGTTCTCTACACTCTTTCCCTACACGAC
P5-index5-F	AATGATACGGCGACCACCGAGATCTACACCTAATCGAACACTCTTTCCCTACACGAC
P5-index6-F	AATGATACGGCGACCACCGAGATCTACACCTAGAACAACACTCTTTCCCTACACGAC
P5-index7-F	AATGATACGGCGACCACCGAGATCTACACTAAGTTCCACACTCTTTCCCTACACGAC
P5-index8-F	AATGATACGGCGACCACCGAGATCTACACTAGACCTAACACTCTTTCCCTACACGAC
P5-index37-F	AATGATACGGCGACCACCGAGATCTACACTATAGCCTACACTCTTTCCCTACACGAC
P5-index38-F	AATGATACGGCGACCACCGAGATCTACACATAGAGGCACACTCTTTCCCTACACGAC
P5-index39-F	AATGATACGGCGACCACCGAGATCTACACCCTATCCTACACTCTTTCCCTACACGAC
P5-index40-F	AATGATACGGCGACCACCGAGATCTACACGGCTCTGAACACTCTTTCCCTACACGAC
P5-index41-F	AATGATACGGCGACCACCGAGATCTACACAGGCGAAGACACTCTTTCCCTACACGAC
P5-index42-F	AATGATACGGCGACCACCGAGATCTACACTAATCTTAACACTCTTTCCCTACACGAC
P5-index43-F	AATGATACGGCGACCACCGAGATCTACACCAGGACGTACACTCTTTCCCTACACGAC
P5-index44-F	AATGATACGGCGACCACCGAGATCTACACGTACTGACACACTCTTTCCCTACACGAC
P7-adapter25-R	CAAGCAGAAGACGGCATACGAGATCGAGTAATGTGACTGGAGTTCAGACGTGTG
P7-adapter26-R	CAAGCAGAAGACGGCATACGAGATTCTCCGGAGTGACTGGAGTTCAGACGTGTG
P7-adapter27-R	CAAGCAGAAGACGGCATACGAGATAATGAGCGGTGACTGGAGTTCAGACGTGTG
P7-adapter28-R	CAAGCAGAAGACGGCATACGAGATGGAATCTCGTGACTGGAGTTCAGACGTGTG
P7-adapter29-R	CAAGCAGAAGACGGCATACGAGATTTCTGAATGTGACTGGAGTTCAGACGTGTG
P7-adapter30-R	CAAGCAGAAGACGGCATACGAGATACGAATTCGTGACTGGAGTTCAGACGTGTG
P7-adapter31-R	CAAGCAGAAGACGGCATACGAGATAGCTTCAGGTGACTGGAGTTCAGACGTGTG
P7-adapter32-R	CAAGCAGAAGACGGCATACGAGATGCGCATTAGTGACTGGAGTTCAGACGTGTG
P7-adapter33-R	CAAGCAGAAGACGGCATACGAGATCATAGCCGGTGACTGGAGTTCAGACGTGTG
P7-adapter34-R	CAAGCAGAAGACGGCATACGAGATTTCGCGGAGTGACTGGAGTTCAGACGTGTG
P7-adapter35-R	CAAGCAGAAGACGGCATACGAGATGCGCGAGAGTGACTGGAGTTCAGACGTGTG
P7-adapter36-R	CAAGCAGAAGACGGCATACGAGATCTATCGCTGTGACTGGAGTTCAGACGTGTG
P7-adapter37-R	CAAGCAGAAGACGGCATACGAGATGTCGTGATGTGACTGGAGTTCAGACGTGTG
P7-adapter38-R	CAAGCAGAAGACGGCATACGAGATACCACTGTGTGACTGGAGTTCAGACGTGTG
P7-adapter39-R	CAAGCAGAAGACGGCATACGAGATTGGATCTGGTGACTGGAGTTCAGACGTGTG
P7-adapter40-R	CAAGCAGAAGACGGCATACGAGATCCGTTTGTGTGACTGGAGTTCAGACGTGTG
P7-adapter41-R	CAAGCAGAAGACGGCATACGAGATTGCTGGGTGTGACTGGAGTTCAGACGTGTG

P7-adapter42-R	CAAGCAGAAGACGGCATACGAGATGAGGGGTTGTGACTGGAGTTCAGACGTGTG
P7-adapter43-R	CAAGCAGAAGACGGCATACGAGATAGGTTGGGGTGACTGGAGTTCAGACGTGTG
P7-adapter44-R	CAAGCAGAAGACGGCATACGAGATGTGTGGTGGTGACTGGAGTTCAGACGTGTG
P7-adapter45-R	CAAGCAGAAGACGGCATACGAGATTGGGTTTCGTGACTGGAGTTCAGACGTGTG
P7-adapter46-R	CAAGCAGAAGACGGCATACGAGATTGGTCACAGTGACTGGAGTTCAGACGTGTG
P7-adapter47-R	CAAGCAGAAGACGGCATACGAGATTTGACCCTGTGACTGGAGTTCAGACGTGTG
P7-adapter48-R	CAAGCAGAAGACGGCATACGAGATCCACTCCTGTGACTGGAGTTCAGACGTGTG

* *KRAS*-NGS-F/R and *FLT3*-NGS-F/R are the primers for the first PCR of NGS. P5 and P7 are different barcoded primers.

Table S6.

Template and primer sequence of *KRAS* G12D/V/R multiple SPEAR assay

purpose	Name	*Sequence (5′ -3′)
The location of *KRAS* G12 mutations and inner control sequence on the *KRAS* gene.	*KRAS*-WT gene	ACTGAATATAAACTTGTGGTAGTTGGAGCTGGTGGCGTAGGC<br>AAGAGTGCCTTGACGATACAGCTAATTCAGAATCATTTTGTG
	KRAS-G12D gene	ACTGAATATAAACTTGTGGTAGTTGGAGCTGATGGCGTAGGC<br>AAGAGTGCCTTGACGATACAGCTAATTCAGAATCATTTTGTG
	KRAS-G12V gene	ACTGAATATAAACTTGTGGTAGTTGGAGCTGTTGGCGTAGGC<br>AAGAGTGCCTTGACGATACAGCTAATTCAGAATCATTTTGTG
	KRAS-G12R gene	ACTGAATATAAACTTGTGGTAGTTGGAGCTCGTGGCGTAGGCA<br>AGAGTGCCTTGACGATACAGCTAATTCAGAATCATTTTGTG
Sequence information for in *vitro* sgRNA transcription	sgRNA-T7-universal	TAATACGACTCACTATAGGG
	AapCas12b-sgRNA	TAATACGACTCACTATAGGGGTCTAGAGGACAGAATTTTT<br>CAACGGGTGTGCCAATGGCCACTTTCCAGGTGGCAAAGCC<br>CGTTGAGCTTCTCAAATCTGAGAAGTGGCACGTAGCAGCA<br>AGATTAGCAGAAGCT
	KRAS-12b-WT-17gR	ACCAGCTCCAACTACCA-GTGCCACTTCTCAGATTTGA
	KRAS-12b-G12V-17gR	AACAGCTCCAACTACCA-GTGCCACTTCTCAGATTTGA
	KRAS-12b-G12D-17gR	ATCAGCTCCAACTACCA-GTGCCACTTCTCAGATTTGA
	KRAS-12b-G12R-17gR	ACGAGCTCCAACTACCA-GTGCCACTTCTCAGATTTGA
	KRAS-12b-IR-17gR	TGAATTAGCTGTATCGT-GTGCCACTTCTCAGATTTGA
SPEAR Primer	*KRAS*-mixF	TGGTTAATGTGGAGTCACTGAATATAAACTTGT
	KRAS-mixR	GCGTATAAGTAGAGTCCACAAAATGATTCTGAA

* The recognition sequence of the *KRAS* G12 sgRNAs are highlighted in yellow, the PAM site in cyan, and the mutation sites are highlighted in red. The recognition sequence of inner control sgRNA is underlined, the PAM site is indicated by wavy line.

Supplementary Note

Note S1. Sequence information of plasmids

The sgRNA recognition sequence is highlighted in yellow, the PAM site in cyan, the target binding sequences of the F and R primers for SPEAR are underlined below, and the mutation site is marked in red.

> *KRAS*-WT gene (499-nt)

TCCTAGGCGGCGGCCGCGGCGGCGGAGGCAGCAGCGGCGGCGGCAGTGGCGGCGGCGAAG
GTGGCGGCGGCTCGGCCAGTACTCCCGGCCCCCGCCATTTCGGACTGGGAGCGAGCGCGGCG
CAGGCACTGAAGGCGGCGGCGGGGCCAGAGGCTCAGCGGCTCCCAGGTGCGGGAGAGAGG
CCTGCTGAAAATGACTGAATATAAACTTGTGGTAGTTGGAGCTGGTGGCGTAGGCAAGAGTG
CCTTGACGATACAGCTAATTCAGAATCATTTTGTGGACGAATATGATCCAACAATAGAGGATTC
CTACAGGAAGCAAGTAGTAATTGATGGAGAAACCTGTCTCTTGGATATTCTCGACACAGCAGG
TCAAGAGGAGTACAGTGCAATGAGGGACCAGTACATGAGGACTGGGGAGGGCTTTCTTTGTG
TATTTGCCATAAATAATACTAAATCATTTGAAGATATTCACCATTATAGAGAACAAATTAAAAGA
GT

> *KRAS*-G12D gene (499-nt)

TCCTAGGCGGCGGCCGCGGCGGCGGAGGCAGCAGCGGCGGCGGCAGTGGCGGCGGCGAAG
GTGGCGGCGGCTCGGCCAGTACTCCCGGCCCCCGCCATTTCGGACTGGGAGCGAGCGCGGCG
CAGGCACTGAAGGCGGCGGCGGGGCCAGAGGCTCAGCGGCTCCCAGGTGCGGGAGAGAGG
CCTGCTGAAAATGACTGAATATAAACTTGTGGTAGTTGGAGCTGATGGCGTAGGCAAGAGTGC
CTTGACGATACAGCTAATTCAGAATCATTTTGTGGACGAATATGATCCAACAATAGAGGATTCC
TACAGGAAGCAAGTAGTAATTGATGGAGAAACCTGTCTCTTGGATATTCTCGACACAGCAGGT
CAAGAGGAGTACAGTGCAATGAGGGACCAGTACATGAGGACTGGGGAGGGCTTTCTTTGTGT
ATTTGCCATAAATAATACTAAATCATTTGAAGATATTCACCATTATAGAGAACAAATTAAAAGAG
T

> *KRAS*-G12V gene (499-nt)

TCCTAGGCGGCGGCCGCGGCGGCGGAGGCAGCAGCGGCGGCGGCAGTGGCGGCGGCGAAG
GTGGCGGCGGCTCGGCCAGTACTCCCGGCCCCCGCCATTTCGGACTGGGAGCGAGCGCGGCG
CAGGCACTGAAGGCGGCGGCGGGGCCAGAGGCTCAGCGGCTCCCAGGTGCGGGAGAGAGG
CCTGCTGAAAATGACTGAATATAAACTTGTGGTAGTTGGAGCTGTTGGCGTAGGCAAGAGTGC

CTTGACGATACAGCTAATTCAGAATCATTTTGTGGACGAATATGATCCAACAATAGAGGATTCC
TACAGGAAGCAAGTAGTAATTGATGGAGAAACCTGTCTCTTGGATATTCTCGACACAGCAGGT
CAAGAGGAGTACAGTGCAATGAGGGACCAGTACATGAGGACTGGGGAGGGCTTTCTTTGTGT
ATTTGCCATAAATAATACTAAATCATTTGAAGATATTCACCATTATAGAGAACAAATTAAAAGAG
T

> *KRAS*-G12R gene (499-nt)

TCCTAGGCGGCGGCCGCGGCGGCGGAGGCAGCAGCGGCGGCGGCAGTGGCGGCGGCGAAG
GTGGCGGCGGCTCGGCCAGTACTCCCGGCCCCCGCCATTTCGGACTGGGAGCGAGCGCGGCG
CAGGCACTGAAGGCGGCGGCGGGGCCAGAGGCTCAGCGGCTCCCAGGTGCGGGAGAGAGG
CCTGCTGAAAATGACTGAATATAAACTTGTGGTAGTTGGAGCTCGTGGCGTAGGCAAGAGTGC
CTTGACGATACAGCTAATTCAGAATCATTTTGTGGACGAATATGATCCAACAATAGAGGATTCC
TACAGGAAGCAAGTAGTAATTGATGGAGAAACCTGTCTCTTGGATATTCTCGACACAGCAGGT
CAAGAGGAGTACAGTGCAATGAGGGACCAGTACATGAGGACTGGGGAGGGCTTTCTTTGTGT
ATTTGCCATAAATAATACTAAATCATTTGAAGATATTCACCATTATAGAGAACAAATTAAAAGAG
T

> *FLT3*-WT gene (499-nt)

TTTCACTCTGAAGATGAAATTGAATATGAAAACCAAAAAAGGCTGGAAGAAGAGGAGGACTT
GAATGTGCTTACATTTGAAGATCTTCTTTGCTTTGCATATCAAGTTGCCAAAGGAATGGAATTT
CTGGAATTTAAGTCGTGTGTTCACAGAGACCTGGCCGCCAGGAACGTGCTTGTCACCCACGG
GAAAGTGGTGAAGATATGTGACTTTGGATTGGCTCGAGATATCATGAGTGATTCCAACTATGTT
GTCAGGGGCAATGCCCGTCTGCCTGTAAAATGGATGGCCCCCGAAAGCCTGTTTGAAGGCAT
CTACACCATTAAGAGTGATGTCTGGTCATATGGAATATTACTGTGGGAAATCTTCTCACTTGGT
GTGAATCCTTACCCTGGCATTCCGGTTGATGCTAACTTCTACAAACTGATTCAAAATGGATTTA
AAATGGATCAGCCATTTTATGCTACAGAAGAAATATACATTATAATGCAATCCTGCT

> *FLT3*-D835Y gene (499-nt)

TTTCACTCTGAAGATGAAATTGAATATGAAAACCAAAAAAGGCTGGAAGAAGAGGAGGACTT
GAATGTGCTTACATTTGAAGATCTTCTTTGCTTTGCATATCAAGTTGCCAAAGGAATGGAATTT
CTGGAATTTAAGTCGTGTGTTCACAGAGACCTGGCCGCCAGGAACGTGCTTGTCACCCACGG
GAAAGTGGTGAAGATATGTGACTTTGGATTGGCTCGATATATCATGAGTGATTCCAACTATGTT
GTCAGGGGCAATGCCCGTCTGCCTGTAAAATGGATGGCCCCCGAAAGCCTGTTTGAAGGCAT
CTACACCATTAAGAGTGATGTCTGGTCATATGGAATATTACTGTGGGAAATCTTCTCACTTGGT

GTGAATCCTTACCCTGGCATTCCGGTTGATGCTAACTTCTACAAACTGATTCAAAATGGATTTA
AAATGGATCAGCCATTTTATGCTACAGAAGAAATATACATTATAATGCAATCCTGCT

> *FLT*3-D835H gene (499-nt)

TTTCACTCTGAAGATGAAATTGAATATGAAAACCAAAAAAGGCTGGAAGAAGAGGAGGACTT
GAATGTGCTTACATTTGAAGATCTTCTTTGCTTTGCATATCAAGTTGCCAAAGGAATGGAATTT
CTGGAATTTAAGTCGTGTGTTCACAGAGACCTGGCCGCCAGGAACGTGCTTGTCACCCACGG
GAAAGTGGTGAAGATATGTGACTTTGGATTGGCTCGACATATCATGAGTGATTCCAACTATGTT
GTCAGGGGCAATGCCCGTCTGCCTGTAAAATGGATGGCCCCCGAAAGCCTGTTTGAAGGCAT
CTACACCATTAAGAGTGATGTCTGGTCATATGGAATATTACTGTGGGAAATCTTCTCACTTGGT
GTGAATCCTTACCCTGGCATTCCGGTTGATGCTAACTTCTACAAACTGATTCAAAATGGATTTA
AAATGGATCAGCCATTTTATGCTACAGAAGAAATATACATTATAATGCAATCCTGCT

> *FLT*3-D835V gene (499-nt)

TTTCACTCTGAAGATGAAATTGAATATGAAAACCAAAAAAGGCTGGAAGAAGAGGAGGACTT
GAATGTGCTTACATTTGAAGATCTTCTTTGCTTTGCATATCAAGTTGCCAAAGGAATGGAATTT
CTGGAATTTAAGTCGTGTGTTCACAGAGACCTGGCCGCCAGGAACGTGCTTGTCACCCACGG
GAAAGTGGTGAAGATATGTGACTTTGGATTGGCTCGAGTTATCATGAGTGATTCCAACTATGTT
GTCAGGGGCAATGCCCGTCTGCCTGTAAAATGGATGGCCCCCGAAAGCCTGTTTGAAGGCAT
CTACACCATTAAGAGTGATGTCTGGTCATATGGAATATTACTGTGGGAAATCTTCTCACTTGGT
GTGAATCCTTACCCTGGCATTCCGGTTGATGCTAACTTCTACAAACTGATTCAAAATGGATTTA
AAATGGATCAGCCATTTTATGCTACAGAAGAAATATACATTATAATGCAATCCTGCT

> *FLT*3-D835F gene (499-nt)

TTTCACTCTGAAGATGAAATTGAATATGAAAACCAAAAAAGGCTGGAAGAAGAGGAGGACTT
GAATGTGCTTACATTTGAAGATCTTCTTTGCTTTGCATATCAAGTTGCCAAAGGAATGGAATTT
CTGGAATTTAAGTCGTGTGTTCACAGAGACCTGGCCGCCAGGAACGTGCTTGTCACCCACGG
GAAAGTGGTGAAGATATGTGACTTTGGATTGGCTCGATTTATCATGAGTGATTCCAACTATGTT
GTCAGGGGCAATGCCCGTCTGCCTGTAAAATGGATGGCCCCCGAAAGCCTGTTTGAAGGCAT
CTACACCATTAAGAGTGATGTCTGGTCATATGGAATATTACTGTGGGAAATCTTCTCACTTGGT
GTGAATCCTTACCCTGGCATTCCGGTTGATGCTAACTTCTACAAACTGATTCAAAATGGATTTA
AAATGGATCAGCCATTTTATGCTACAGAAGAAATATACATTATAATGCAATCCTGCT

> *MYD*88-WT gene (499-nt)

TGAGGGAATGTGTAGGTGGGGCCTCTGGATTGTCAGCCTTCCCTCCCCAAGGACTGTGGATGC
AGTACCAAAGAACTGCTGAAGATCTCTGCACACCTGAGCATGTGTGCATGTGTGTGCCTTTTT
GTGTGAGTGAATGTGTGCCAGGGGTACTTAGATGGGGGATGGCTGTTGTTAACCCTGGGGTTG
AAGACTGGGCTTGTCCCACCATGGGGCAAGGGCCTGATGCCAGCATGGCACCCCTTGGCTTG
CAG<u>GTGCCCATCAGAAGCGA</u>CTGATCCCCATCAAGTACAA<u>GGCAATGAAGAAAGAGT</u>TCCCC
AGCATCCTGAGGTTCATCACTGTCTGCGACTACACCAACCCCTGCACCAAATCTTGGTTCTGG
ACTCGCCTTGCCAAGGCCTTGTCCCTGCCCTGAAGACTGTTCTGAGGCCCTGGGTGTGTGTGT
ATCTGTCTGCCTGTCCATGTACTTCTGCCCTGCCTCCTCCTTTCGTTGTAGGAGGAATCT

> *MYD*88-L265P gene (499-nt)

TGAGGGAATGTGTAGGTGGGGCCTCTGGATTGTCAGCCTTCCCTCCCCAAGGACTGTGGATGC
AGTACCAAAGAACTGCTGAAGATCTCTGCACACCTGAGCATGTGTGCATGTGTGTGCCTTTTT
GTGTGAGTGAATGTGTGCCAGGGGTACTTAGATGGGGGATGGCTGTTGTTAACCCTGGGGTTG
AAGACTGGGCTTGTCCCACCATGGGGCAAGGGCCTGATGCCAGCATGGCACCCCTTGGCTTG
CAG<u>GTGCCCATCAGAAGCGA</u>CCGATCCCCATCAAGTACAA<u>GGCAATGAAGAAAGAGT</u>TCCCC
AGCATCCTGAGGTTCATCACTGTCTGCGACTACACCAACCCCTGCACCAAATCTTGGTTCTGG
ACTCGCCTTGCCAAGGCCTTGTCCCTGCCCTGAAGACTGTTCTGAGGCCCTGGGTGTGTGTGT
ATCTGTCTGCCTGTCCATGTACTTCTGCCCTGCCTCCTCCTTTCGTTGTAGGAGGAATCT
